# Targeting WEE1 to enhance conventional therapies for acute lymphoblastic leukemia

**DOI:** 10.1186/s13045-018-0641-1

**Published:** 2018-08-01

**Authors:** Andrea Ghelli Luserna Di Rorà, Neil Beeharry, Enrica Imbrogno, Anna Ferrari, Valentina Robustelli, Simona Righi, Elena Sabattini, Maria Vittoria Verga Falzacappa, Chiara Ronchini, Nicoletta Testoni, Carmen Baldazzi, Cristina Papayannidis, Maria Chiara Abbenante, Giovanni Marconi, Stefania Paolini, Sarah Parisi, Chiara Sartor, Maria Chiara Fontana, Serena De Matteis, Ilaria Iacobucci, Pier Giuseppe Pelicci, Michele Cavo, Timothy J. Yen, Giovanni Martinelli

**Affiliations:** 1 Department of Experimental, Diagnostic and Specialty Medicine, Institute of Hematology “L. e A. Seràgnoli”, University of Bologna, Via Massarenti 9, 40138 Bologna, Italy; 2 Laboratory of Clinical Genomics, European Institute of Oncology, Milan, Italy; 3 Cancer Biology Program, Fox Chase Cancer Center, Philadelphia, PA USA; 4 Biosciences Laboratory, Istituto Scientifico Romagnolo per lo Studio e la Cura dei Tumori (IRST) IRCCS, Meldola, Italy; 5 Istituto Scientifico Romagnolo per lo Studio e la Cura dei Tumori (IRST) IRCCS, Meldola, Italy; 6 LAM Therapeutics, Guilford, CT USA; 7 Department of Pathology, St. Jude Children’s Research Hospital, Memphis, TN USA

**Keywords:** Acute lymphoblastic leukemia, WEE1 inhibitor, Chemo-sensitizer agent, G2/M checkpoint

## Abstract

**Background:**

Despite the recent progress that has been made in the understanding and treatment of acute lymphoblastic leukemia (ALL), the outcome is still dismal in adult ALL cases. Several studies in solid tumors identified high expression of WEE1 kinase as a poor prognostic factor and reported its role as a cancer-conserving oncogene that protects cancer cells from DNA damage. Therefore, the targeted inhibition of WEE1 kinase has emerged as a rational strategy to sensitize cancer cells to antineoplastic compounds, which we evaluate in this study.

**Methods:**

The effectiveness of the selective WEE1 inhibitor AZD-1775 as a single agent and in combination with different antineoplastic agents in B and T cell precursor ALL (B/T-ALL) was evaluated in vitro and ex vivo studies. The efficacy of the compound in terms of cytotoxicity, induction of apoptosis, and changes in gene and protein expression was assessed using different B/T-ALL cell lines and confirmed in primary ALL blasts.

**Results:**

We showed that *WEE1* was highly expressed in adult primary ALL bone marrow and peripheral blood blasts (*n* = 58) compared to normal mononuclear cells isolated from the peripheral blood of healthy donors (*p* = 0.004). Thus, we hypothesized that WEE1 could be a rational target in ALL, and its inhibition could enhance the cytotoxicity of conventional therapies used for ALL. We evaluated the efficacy of AZD-1775 as a single agent and in combination with several antineoplastic agents, and we elucidated its mechanisms of action. AZD-1775 reduced cell viability in B/T-ALL cell lines by disrupting the G2/M checkpoint and inducing apoptosis. These findings were confirmed in human primary ALL bone marrow and peripheral blood blasts (*n* = 15). In both cell lines and primary leukemic cells, AZD-1775 significantly enhanced the efficacy of several tyrosine kinase inhibitors (TKIs) such as bosutinib, imatinib, and ponatinib, and of chemotherapeutic agents (clofarabine and doxorubicin) in terms of the reduction of cell viability, apoptosis induction, and inhibition of proliferation.

**Conclusions:**

Our data suggest that WEE1 plays a role in ALL blast’s survival and is a bona fide target for therapeutic intervention. These data support the evaluation of the therapeutic potential of AZD-1775 as chemo-sensitizer agent for the treatment of B/T-ALL.

**Electronic supplementary material:**

The online version of this article (10.1186/s13045-018-0641-1) contains supplementary material, which is available to authorized users.

## Background

Although many progresses have been made in understanding the pathogenesis of ALL and in improving outcome, response rates are still unsatisfied in adult patients with a 5-year survival rate of less than 35%. To date, the therapeutic options, with the exclusion of drugs against particular genetic alterations (*BCR*-*ABL1* fusion or *BCR*-*ABL1*-like alterations), are still mainly based on conventional chemotherapy especially for the induction phase [1–11]. Therefore, there is a clinical need to identify novel targets for more effective therapies and/or improve the effectiveness of the conventional treatments in order to increase the survival rates of adult patients with ALL. It is well established that eukaryotic cells respond to DNA damage by activating specific pathways, collectively termed DNA damage response (DDR) [12–14]. DDR, therefore, refers to a network of biological processes that are activated by aberrant DNA structures generated upon DNA damage, including cell cycle checkpoints, DNA repair mechanisms, cell death, and senescence [15]. The WEE1 kinase is a key player in the DDR process and acts to inhibit mitotic entry in cells with damaged DNA. This is achieved by WEE1-mediated inhibitory phosphorylation on key residues of CDK1 and 2 kinases [16–20]. Several studies speculated on the importance of WEE1 expression in cancer cells ascribing a dual biological role as a tumor suppressor, whose loss promotes the accumulation of genetic aberrations on pre-neoplastic lesions, or as a cancer-conserving oncogene, whose expression protects cancer cells from DNA damage and aberrant mitosis [21–25]. Moreover, different cancer cells depend on the expression of WEE1 for survival as shown by the effectiveness of a selective WEE1 inhibitor [26]. Due to the above-mentioned crucial biological roles, and the relationship between high expression of *WEE1* and poor prognosis in several kinds of tumors [25, 27], selective WEE1 inhibitors (PD0166285, PD0407824, and AZD-1775) have been developed [26, 28–37]. Several preclinical and clinical studies (clinicaltrials.gov; NCT02341456; NCT03012477; NCT03315091; NCT01748825), mostly focused on solid tumors, demonstrated the efficacy of AZD-1775 not only as a single agent but also in combination with DNA damaging drugs or different targeted inhibitors in several cancer models [37–39]. Several studies demonstrate that AZD-1775 is a powerful approach to override chemoresistance in different tumor models. For instance, it has been shown that AZD1775 increased the sensitivity to cisplatin and gemcitabine (both DNA damaging agents) by overriding the G2/M checkpoint and force cancer cells with defective DNA replication to inappropriately enter mitosis and die via mitotic catastrophe [40, 41]. Combinatorial studies can be used to exploit tumor resistance to AZD-1775. Indeed, AZD1775-resistant small cell lung cancer models were shown to have elevated expression of AXL, pS6, and MET genes that a WEE1/AXL or WEE1/mTOR inhibitor combination could overcome the resistance in vitro and in vivo [42]. Despite the promising data from studies using solid tumor models, few studies have investigated the mechanisms of the action of AZD-1775 and its efficacy in hematological malignancies especially in acute leukemia [35–38]. In the present study, we provide evidence that WEE1 represents a rational therapeutic target in ALL. First, we evaluated the levels of expression of *Wee1* mRNA in a cohort of 58 ALL primary samples, and then the effectiveness of AZD-1775, as monotherapy and in combination with different drugs normally used as a standard of care for adult ALL patients.

## Methods

### Drugs and cell lines

AZD-1775 was purchased from MedChemexpress. Clofarabine, doxorubicin, imatinib, and ponatinib were obtained from Sigma-Aldrich. Bosutinib (Bos) was purchased from Tocris, and Bosutinib isomer (Bos-I) was purchased from LC Labs. Human B and T cell precursor ALL (B/T-ALL) cell lines (B-ALL: BV-173, SUP-B15, REH, NALM-6, NALM-19; T-ALL: MOLT-4, RPMI-8402, CCRF-CEM) were cultured in RPMI-1640 (Invitrogen) with 1% l-glutamine (Sigma-Aldrich), penicillin (100 U/ml, Gibco), and streptomycin (100 μg/ml, Gibco) supplemented with 10–20% fetal bovine serum (FBS, Gibco). All the cell lines were purchased from Deutsche Sammlung von Mikroorganismen and Zellkulturen GmbH (DSMZ) website (https://www.dsmz.de).

### Primary leukemic cells and treatment

To assess the effect of AZD-1775 in primary samples, upon written informed consent, primary leukemic cells with > 70% of blasts were isolated from the peripheral blood and bone marrow of adult ALL cases (*n* = 15, Additional file 1: Table S1) and treated ex vivo with increasing concentration of the test drug. The study was performed in accordance with the principles laid down in the Declaration of Helsinki. Primary cells Lymphoprep-isolated (Nycomed UK, Birmingham) were seeded in 6-well plates at 1 × 10^6^ cells/ml in RPMI-1640 supplemented with 20% FBS and treated with AZD-1775 (2.5, 5, and 10 μM) for 24 h. In order to evaluate the cytotoxicity of the compound on non-leukemic cells, mononuclear cells (MNCs) isolated, upon written informed consent, from the peripheral blood of healthy donors (*n* = 5) were incubated in 6-well plates at 1 × 10^6^ cells/ml in RPMI-1640 with 20% FBS and treated with AZD-1775 at the same concentration reported above for 24 h. For drug combination studies, cells were incubated with increasing concentrations of AZD-1775 and a fixed dose of clofarabine (500 nM) or Bos (1 μM) for 24 h. The number of viable cells was detected by trypan blue exclusion dye (Sigma-Aldrich).

### Immunohistochemistry analysis

Bone marrow specimens were fixed in B5 solution for 2 h, soaked in 70% alcohol for at least 30 min, and then decalcified in an EDTA-based solution for 2.5 h. Sections of 3 μm thickness were cut for histological examination (H&E, Giemsa, Gomori silver impregnation) and immunohistochemistry. For diagnostic purpose, the following antibodies were applied in all cases: anti-CD79a (clone JCB, dilution 1:50, DakoCytomation, Glostrup, Denmark), anti-PAX5 (clone DAK-Pax5, dilution 1:60, DakoCytomation), anti-CD20 (clone L26, dilution 1:300, DakoCytomation), anti-CD10 (clone 56C6, dilution 1:50, Leica Biosystem), anti-CD34 (clone QBEnd/10, dilution 1:100, Leica Biosystem), and anti-TdT (clone EP266, dilution 1:40, DakoCytomation). Antigens were retrieved with the PT-link (PT100/PT101, DakoCytomation) and the EnVision Flex Target Retrieval Solution High pH (K8004, DakoCytomation) at 92 °C or 80 °C for 5 min. For the study, the Wee1 antibody was applied in 7/58 bone marrow biopsies available at the Unit of Haemolymphopathology, Bologna. This antibody (clone B-11, 1:30, Santa Cruz Biotechnology, CA, USA) was applied on pre-exposing slides soaked in a Tris-EDTA pH 9 solution at 1 min heating in a pressure cooker. Immunohistochemistry was performed on an Autostainer Plus platform (DakoCytomation), incubating primary antibody at room temperature for 30 min; the reaction was detected by the Dako Real Detection Systems Alkaline Phosphatase/RED Rabbit/Mouse Kit (K 5005, DakoCytomation). Double staining was performed using the Dako Real Detection Systems Alkaline Phosphatase/RED Rabbit/Mouse Kit (K 5005, DakoCytomation) to reveal anti-CD79 and the Dako Real EnVision Detection System, Peroxidase/DAB, Rabbit/Mouse (K5007, DakoCytomation) to highlight anti-Wee1. All slides were counterstained with Gill’s hematoxylin.

### Cell viability and cell proliferation assay

In order to assess cell viability, cells were seeded into 96-well plates at 0.5 × 10^6^ cells/ml and incubated with AZD-1775 (6 to 5000 nM, dilution 1:3 media) for 24, 48, and 72 h. Cell viability was then determined using MTS Cell Proliferation Assay Kit (Promega). For the drug combination index assay, cells were treated simultaneously with increasing concentration of the two test drugs for 24, 48, and 72 h. The additive, synergistic, and antagonistic effect of the drug combinations was evaluated using Compusyn Software where combination index (CI) < 1 synergism, CI = 1 additivity, and CI > 1 antagonism. To assess the proliferation ability, cell lines were seeded in 6-well plates at a concentration of 0.2 × 10^6^ cells/ml and counted every 24 h for 4 days of continuous drug exposure. All drug treatments were performed in triplicate, and independent experiments were performed at least three times.

### Light and fluorescence microscopy analyses

To investigate potential macroscopic modifications of cell morphology, cells (primary and cell lines) were seeded in 6-well plates at 0.5 × 10^5^ cells/ml and incubated with increasing concentration of AZD-1775 for 24 h. Cells were harvested, spun down (10 min at 200 g) onto glass slips using a Cytospin™ centrifuge (Thermofisher), and then stained with the May-Grünwald Giemsa solutions (Sigma-Aldrich). The slides were analyzed using an optical microscope AXIOVERT 40 CFL and the pictures analyzed using AxioVision Rel.4.7 software. For the immunofluorescence analysis, BV-173 cells were seeded to poly-d-lysine-coated coverslips, fixed with 4% paraformaldehyde (PFA) and stained at 37 °C with an anti-phospho-Ser/Thr-Pro MPM-2 antibody FITC conjugated (Millipore Sigma). Coverslips were, then, mounted on glass slides using a mounting media with DAPI (4′,6-diamidino-2-phenylindole) (Prolong Gold with DAPI, Invitrogen). Immunofluorescence analyses were performed using the AXIOVERT 40 CFL microscope and the picture analyzed using AxioVision Rel.4.7 software.

### Flow cytometry

All analyses were performed using the flow cytometer Facs CantoII (BD). Apoptosis was performed using Annexin V/propidium iodide (PI) according to the manufacturer’s instructions (Roche). Cells were seeded in 12-well plates at 0.5 × 10^5^ cells/ml with AZD-1775 (at IC_50_, IC_25_, and IC_12.5_) for 24 h at 37 °C. The percentage of Annexin V/PI-positive cells was determined by assaying a minimum of 10,000 cells. The mean percentage of Annexin V/PI-positive cells and standard error measurements were calculated from at least two independent experiments. Cell cycle analyses were performed using the PI staining mix (BD). Cells were seeded in a 24-well plate at a concentration of 0.5 × 10^6^ cells per well and treated with AZD-1775 (IC_50_) for 24 h. After 24 h of incubation, the cells were harvested and washed with cold PBS. After washing with PBS, the cells were fixed using ethanol 70% and stored at − 20 °C for 24 h. After the fixation period, the ethanol was removed by one wash in PBS, and the cells were incubated for 30 min at 37 °C with the PI staining mix. The quantitative analyses were performed using Flowing and ModfiT software (Verity Software House).

### Immunoblotting

Immunoblotting analyses were performed using Mini-Protean TGX stain-free precast gels, blotted to nitrocellulose membranes (Bio-Rad Trans-blot turbo transfer pack) and incubated overnight with the following antibodies: Chk1 (#2345S), pChk1 (Ser317) (#2344S), pChk1 (ser296) (#2349), Cdc2 (#9112S), pCdc2 (Tyr15) (#4539S), Cdc25C (#4688), Cdc25B (#9525), CCNB1 (#4138), CDKN1A (#2947), pH2A.X (Ser139) (#2577S), MYT1 (#4282), p-c-ABL (Tyr245) (#2868), and Rad51 (#8875) from Cell Signaling. Antibody to CCNB2 (ab18250) was from Abcam. Antibody to β-actin was from Sigma (St. Louis, MO). Finally, all the antibodies were detected using the enhanced chemiluminescence kit ECL (GE) and the compact darkroom ChemiDoc-It (UVP).

### Quantitative PCR and gene expression

To evaluate how AZD-1775 affects the gene expression of different components of the G2/M checkpoint, cell lines (BV-173 and CCRF-CEM) and primary cells were treated for 12 h with increasing concentration of AZD-1775 at approximately their IC_50_. The treatment was performed for 12 h in order to highlight the effect of the compound on gene expression before inducing overt cytotoxicity. After the period of incubation, the cells were harvested and the total RNA was extracted using Maxwell simply RNA Blood kit (Promega), and 1 μg of each RNA sample (quantified by ND1000 Spectrophotometer) was reverse transcribed using iScript Advanced cDNA Synthesis kit for RT-qPCR (Bio-Rad). For the quantification of G2/M checkpoint genes, the commercial 96-well PrimePCR plates (DNA damage DNA-ATM/ATR regulation of G2/M checkpoint, Bio-Rad) were employed according to the instructions of the manufacturer: SsoAdvanced Universal Sybr Green Supermix (Bio-Rad) and LightCycler 480 System amplification protocol (Roche Diagnostics, Mannheim, Germany). Data analysis was performed with PrimePCR analysis software (Bio-Rad).

### Gene expression profile

Gene expression profiling on 58 adult ALL patient samples (Additional file 1: Table S1) and on 7 MNCs samples obtained from the peripheral blood of seven healthy donors was performed using Affymetrix GeneChip Human Transcriptome Array 2.0 (Affymetrix Inc., Santa Clara, CA, USA; currently ThermoFisher Scientific) following the manufacturers’ instructions. In particular, we analyzed 33 *BCR*-*ABL1*-negative (27 at the time of diagnosis and six unpaired relapses) and 25 *BCR*-*ABL1*-positive (17 at the time of diagnosis, eight unpaired relapses) samples. Raw data were normalized with Expression Console Software 1.4 (Affymetrix Inc.; currently ThermoFisher Scientific) by using the gene-level SST RMA algorithm.

### Statistical analysis

Data were presented as the mean ± standard deviation (SD) from at least three biological replicates. Two-tailed *t* test or one-way ANOVA test were used to analyze the statistical significance between the groups. *p* value < 0.05 was considered a significant difference. Statistical analysis was performed with Graphpad5 software (GraphPad Inc., San Diego, CA, USA).

## Results

### *Wee1* transcript is highly expressed in ALL primary samples

**Fig. 1:**
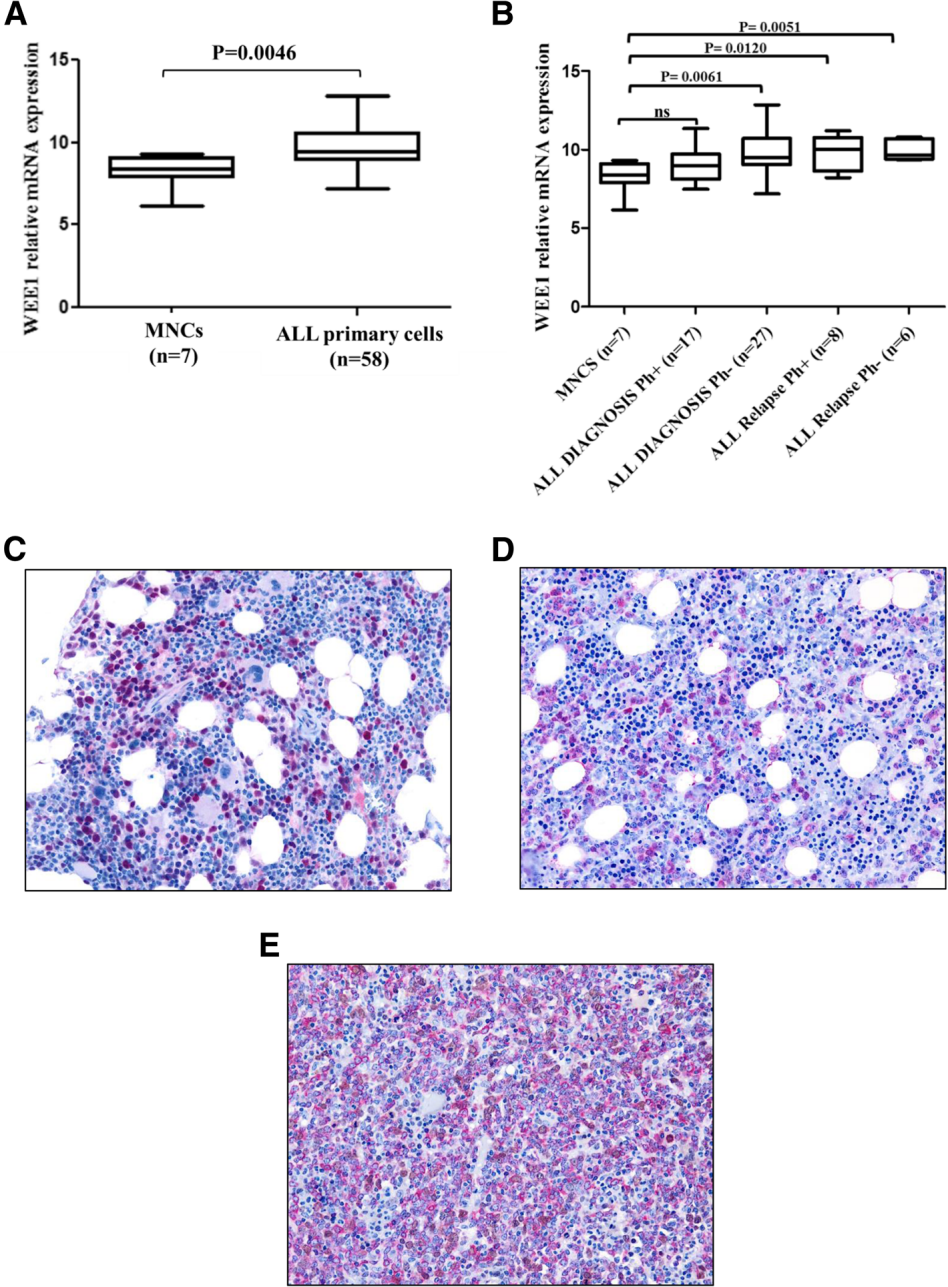
WEE1 overexpression in ALL samples. **a** WEE1 transcript levels in samples isolated from adult ALL (*n* = 58) and in MNCs (*n* = 7) from peripheral blood of healthy donors. One-way ANOVA test was performed to confirm the statistical significance of the differences. Results are expressed as Log10 2exp[−(ΔΔCt). **b** WEE1 transcript levels in samples isolated from adult *BCR*-*ABL1*-positive (Ph+) ALL at diagnosis (*n* = 17), adult *BCR*-*ABL1*-negative (Ph−) ALL at diagnosis (*n* = 27), adult *BCR*-*ABL1*-positive ALL at relapse (unpaired, *n* = 8), adult *BCR*-*ABL1*-negative ALL at relapse (unpaired, *n* = 6), and in MNCs (*n* = 7) from the peripheral blood samples of healthy donors. Results are expressed as Log10 2exp[−(ΔΔCt). **c** Immunohistochemistry analysis of a reactive bone marrow sample; WEE1 is positive at moderate to strong intensity in morphologically typical myeloid precursors (× 20). **d** Immunohistochemistry analysis of leukemic blasts scattered in the interstitium and positive for WEE1 (× 20). **e** Leukemic blasts are widely positive for the B cell marker CD79a (red) while those positive for WEE1 (brown) are much fewer, as shown by the few scattered double-stained blasts (× 20)

Gene expression analysis revealed that *Wee1* is highly expressed in adult ALL samples (*n* = 58, 44 at diagnosis and 14 at unpaired relapses) compared to normal mononuclear cells (MNCs) (*p* = 0.0046) (Fig. 1a). Among different leukemia subtypes, *Wee1* was significantly higher in *BCR*-*ABL1*-negative samples (diagnosis *p* = 0.0061; relapse *p* = 0.005) than in *BCR*-*ABL1*-positive samples (diagnosis ns; relapse *p* = 0.01) compared with normal MNCs (Fig. 1b) (Additional file 1: Table S1). To correlate the level of *Wee1* transcript with its protein levels, immunohistochemistry analyses were performed on eight ALL samples. In the bone marrow biopsies, all cases were confirmed by immunohistochemistry as B cell precursor lymphoblastic leukemias (B-ALL); upon CD20 reactivity, three cases were staged as pro-B-ALL (CD20-negative) and four as pre-B-ALL (CD20-positive). Regarding the WEE1 staining, in the reactive bone marrow sections, the protein revealed moderate to strong nuclear and/or nuclear-cytoplasmic staining in a subset of cells morphologically referable as myeloid immature precursors and, more rarely, in the nuclei of the megakaryocytes (Fig. 1c). In the neoplastic blasts, the WEE1 protein localized in the nuclear and/or nuclear-cytoplasmic compartments. The percentage of positive cells was defined in relation to the comparison of CD79a staining in leukemic blasts. Two bone marrow samples turned out to be negative for WEE1 staining on leukemic blasts (ALL_34 and ALL_52); while in the remaining five samples, the percentage of positive blasts ranged from 20% (ALL_26 and ALL_47) to 50% (ALL_35) and to more than 75% (ALL_48 and ALL_22) (Fig. 1d) of the blastic population. In the two cases with 20% positivity, a double staining WEE1/CD79a was performed to correctly assess the amount of double-stained cells (Fig. 1e). These results suggest that given the association of WEE1 abundance in ALL relative to normal cells, WEE1 can be considered a logical therapeutic target.

### AZD-1775 reduces cell viability and induces apoptosis in ALL cell lines

**Fig. 2:**
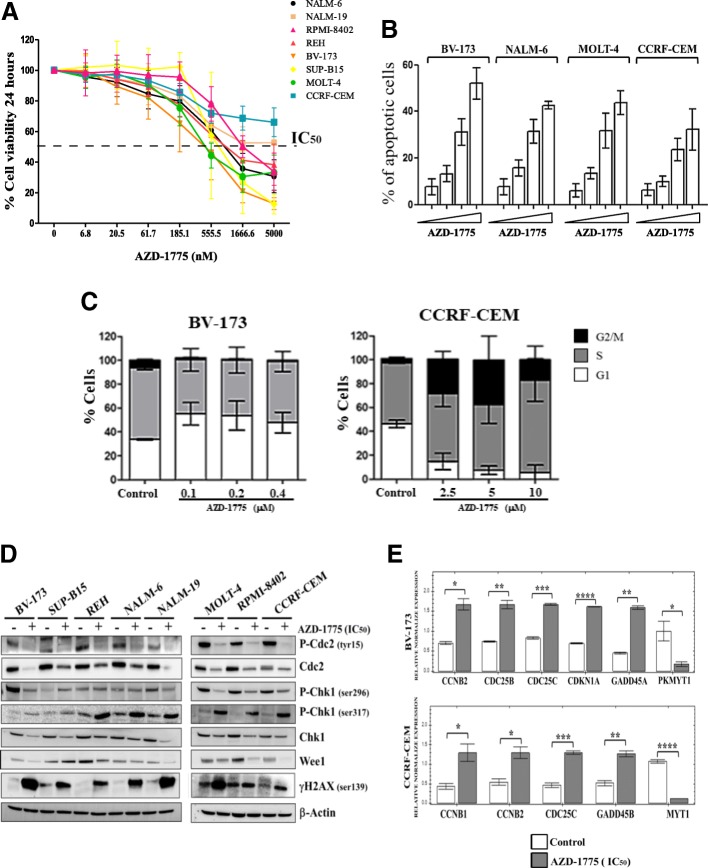
AZD-1775 overrides the G2/M checkpoint and induces mitosis in B/T-ALL cell lines. **a** Viability analyses in ALL cell lines incubated for 24 h with AZD-1775 (6 to 5000 nM). The percentage of viable cells is depicted as a percentage of untreated controls. **b** Apoptosis analyses in BV-173, NALM-6, MOLT-4, and CCRF-CEM cells after 24 h of incubation with AZD-1775 (for each cell line: IC_50_, IC_25_, and IC12.5). The percentage of apoptotic cells was detected after Annexin V/propidium iodide staining. **c** Cell cycle analysis of BV-173 and CCRF-CEM cell line incubated for 24 h with increasing concentration of AZD-1775. **d** Representative immunoblots showing the expression of key proteins of the WEE1 pathway after treatment with AZD-1775 (IC_50_ for each cell line) for 24 h of the indicated cells lines. β-actin was used for loading normalization. **e** Quantitative mRNA analysis of six representative genes of 24 G2/M checkpoint genes analyzed. The white columns represent the controls and the gray columns represent the samples treated with AZD-1775 (IC_50_) for 12 h of the indicated cell lines. Results are expressed as Log10 2exp[−(ΔΔCt)

Having established that the abundance of WEE1 is significantly higher in primary ALL blasts than normal MNCs, this raised the possibility that ALL cells may be reliant on WEE1 for survival. We therefore evaluated the efficacy of a selective WEE1 inhibitor (AZD-1775) on ALL cell lines. AZD-1775 as a single agent reduced cell viability in a dose- and time-dependent manner in a panel of eight ALL cell lines (Fig. 2a, Additional file 2: Figure S1A and B). Consistent with our previous results using inhibitors that target Chk1 and Chk2 checkpoint kinases [43, 44], the sensitivity to AZD-1775 did not correlate with leukemia subtypes, karyotype, or with the basal expression of WEE1 (data not shown). We next treated BV-173 (*BCR*-*ABL1*-positive B-ALL), NALM-6 (*BCR*-*ABL1*-negative B-ALL), MOLT-4 (T-ALL), and CCRF-CEM (T-ALL) cells with AZD-1775 for 24 h at the IC_50_, IC_25_, and IC_12.5_ for each cell line and determined the percentage of apoptotic cells using flow cytometry. In all cell lines, AZD-1775 induced apoptosis in a dose-dependent manner (Fig. 2b).

### AZD-1775 changes the cell cycle profile and activates the DNA damage response pathway in ALL cell lines

Given the known role of WEE1 in cell cycle regulation, we next determined whether AZD-1775 adversely affected cell cycle progression. Cell cycle analysis showed that the treatment with AZD-1775 (IC_50_) for 24 h increased the percentage of cells in G1 phase in BV-173 cell line. Conversely, in CCRF-CEM, we observed a progressive increase in the percentage of cells accumulating in S and G2/M (Fig. 2c). A shorter treatment (12 h) highlighted that in BV-173, AZD-1775 increased the percentage of cells in S phase whereas in CCRF-CEM the percentage in S and G2/M (Additional file 2: Figure S1c). Immunoblotting analysis was conducted to investigate the effects of AZD-1775 on the cell cycle checkpoint and on the DNA damage induction. AZD-1775 treatment reduced phospho-CDC2 Tyr^15^, confirming the inhibition of WEE1 activity [45], and also reduced the basal amount of the protein in all treated samples. Since the G2/M checkpoint is mostly regulated by the activity of CHK1, which cooperates with WEE1 in the regulation of the activity of CDC2-Cyclin B1 complex [45], the expression of this kinase was evaluated in all samples. The expression of phospho-CHK1 Ser^317^, a marker of CHK1 activation in response to DNA damage, was increased in all treated cells while the expression of the total abundance of CHK1 was heterogeneously modified (Fig. 2d). To better understand how AZD-1775 impairs the G2/M checkpoint, quantitative PCR analysis of 24 genes involved in the G2/M checkpoint was performed on the most sensitive (BV-173) and less sensitive (CCRF-CEM) cell lines incubated for 12 h with AZD-1775 at the previously established IC_50_. In both cell lines, *CCNB2* (*p* = 0.05) and *CDC25C* (*p* = 0.001) were significantly upregulated while *MYT1* (*PKMYT1*) was significantly downregulated (BV-173 *p* = 0.05; CCRF-CEM *p* = 0.0001). Moreover, while both cell lines showed an increase in genes involved in cell cycle and apoptosis, the specific genes were different: in BV-173 cells, the treatment upregulated the expression of *CDKN1A* (cell cycle) and *GADD45A* (apoptosis), while in CCRF-CEM, the treatment upregulated the expression of *CCNB2* (cell cycle) and *GADD45B* (apoptosis) (Fig. 2e). Immunoblotting analyses of BV-173 cells partially confirmed the changes showed by the gene expression analyses. Indeed, AZD-1775 upregulated the protein expression levels of CDKN1A, CDC25C, CDC25B, and CCNB2, but no reduction of MYT1 was detected (Additional file 2: Figure S1C). Interestingly, in BV-173 cells, the treatment increased the amount of the mitotic isoform of MYT1, but this data was not associated with a concomitant increase of cells in G2/M phase, as observed from the cell cycle analysis. Finally, in contrast to the effect on GADD45A transcript, the abundance of protein was reduced upon drug treatment (Additional file 2: Figure S1C). We correlated the effect on gene transcription with the perturbation on cell cycle profile in BV-173 and CCRF-CEM after 12 h of treatment (IC_50_). For both cell lines, the treatment induced a slight increase in the S phase cells. Only CCRF-CEM cells exhibited a significant increase in cells in the G2/M phase. The G2/M phase delay of CCRF-CEM cells is consistent with the upregulation of transcripts involved in the cell cycle regulation such as *CCNB1*, *CCNB2*, and *CDC25C*. For BV-173 cells, the increase in the S phase cells was confirmed by the upregulation of *CDC25B* which act on late S phase prior to CDC25C for the induction of the G2 transition [46, 47](Additional file 2: Figure S1D). Finally, in order to confirm the induction of DNA damage and to evaluate the potential mechanism of cell death, different immunofluorescence analyses were performed looking at the marker of DNA damage, phospho-γH2AX, and the marker of mitosis, phospho-MPM2. The MPM2 antibody recognizes a phospho-amino acid-containing epitope (peptides containing LTPLK and FTPLQ domains) present on more than 50 proteins of M phase eukaryotic cells [48]. Cells treated with AZD-1775 showed a significantly greater number of apoptotic bodies positive for MPM2, suggesting that apoptosis occurred during mitosis (Additional file 2: Figure S1E).

### AZD-1775 reduces the cell viability of primary leukemic samples

**Fig. 3:**
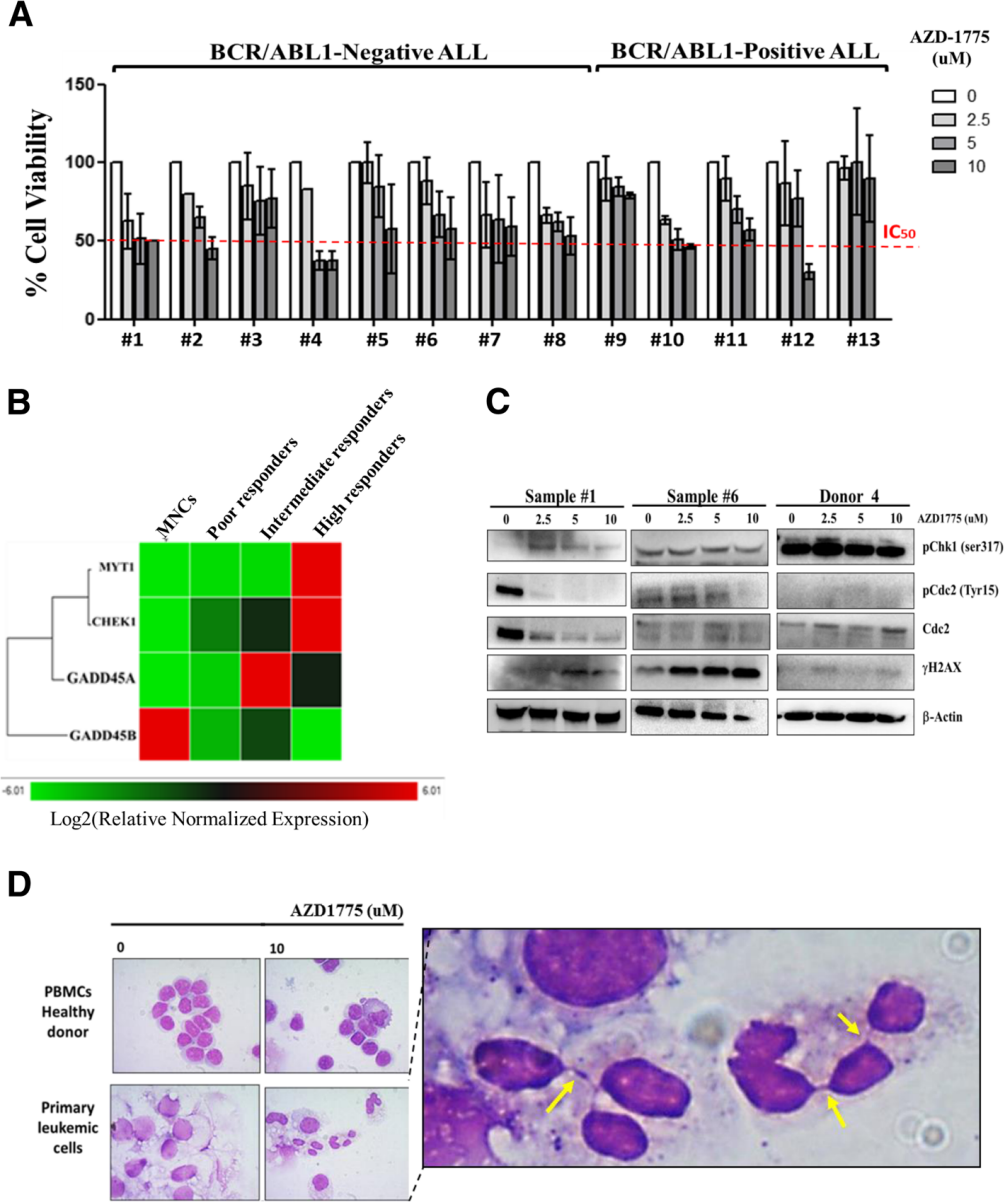
AZD-1775 reduces the cell viability of primary leukemic samples. **a** Cell viability analysis on primary leukemic cells isolated from 13 adult ALL patients treated with AZD-1775 (2.5, 5, and 10 uM) for 24 h. Viable cells are depicted relative to the untreated controls. **b** Quantitative mRNA analyses of 24 genes of the G2/M checkpoint. The basal gene expression of MNCs samples (*n* = 3) was compared with the basal gene expression of poor (*n* = 3), intermediate (*n* = 2), and high (*n* = 2) responders to AZD-1775 (ex vivo). Clustergram with a color indicative of the degree of upregulation (red) or downregulation (green). Targets with similar regulation cluster together. **c** Immunoblotting analyses of primary leukemic cells (*n* = 2, samples #1 and #6) and MNCs (*n* = 1, donor 4) treated with AZD-1775 (2.5, 5, and 10 uM) for 24 h and then stained for markers of WEE1 functional inhibition (phospho-CDC2) and induction of DNA damages (phospho-CHK1 and phospho-γH2AX). β-actin was used for loading normalization. **d** Light microscopy analysis of normal MNCs and primary leukemic cells treated with AZD-1775 (10uM) for 24 h and then stained with May-Grünwald Giemsa solutions. In the figure, the yellow arrows indicate DNA bridges induced by the treatment. Controls in all panels are cells treated with DMSO 0.1%. Representative images are shown at × 100 magnification

The efficacy of AZD-1775 used at 2.5, 5, and 10 μM for 24 h was then evaluated ex vivo on primary leukemic cells isolated from the bone marrow and peripheral blood of adult ALL patients (*n* = 13, Additional file 1: Table S2) and on normal MNCs isolated from the peripheral blood samples of healthy donors (*n* = 5). AZD-1775 reduced the cell viability in a dose-dependent manner in all primary samples but did not affect the viability of normal MNCs (Fig. 3a, Additional file 2: Figure S1F). Despite the small number of samples tested, the sensitivity to AZD-1775 as a single agent apparently did not correlate with the leukemic subtypes (*BCR*-*ABL1*-positive versus *BCR*-*ABL1*-negative ALL patients) nor with the progression of the disease (diagnosis versus relapses). The ex vivo response to AZD-1775 of six primary leukemic samples was correlated with the basal expression of the 24 genes involved in the G2/M checkpoint. The samples were divided into three groups: high responders (IC_50_ within 5 μM), intermediate (IC_50_ within 10 μM), and poor (IC_50_ > 10 μM), and the gene expression profile was compared with that from MNCs. Analysis revealed that three genes were significantly overexpressed in the high responders group in comparison with the other three groups: *CHK1* (*p* = 0.02), *GADD45A* (*p* = 0.01), and *MYT1* (*p* = 0.004) (Fig. 3b, Additional file 1: Table S3). We evaluated the expression of phospho-CHK1 Ser^317^, phospho-CDC2 Tyr^15^, CDC2, and γ-H2AX in two ALL cases (1# and 6#) and the MNCs of one healthy donor (donor 4) after treatment with AZD-1775 at the same concentration reported above. The inhibitor reduced the expression of phospho-CDC2 Tyr^15^ while increased the amount of γH2AX in the leukemic samples but not on normal cells (only a mild increase of γH2AX was observed over baseline). Surprisingly, the treatment induced an increase of phospho-CHK1 Ser^317^ expression in sample 1# and in donor 4 but not in sample 6# (Fig. 3d). In addition, AZD-1775 significantly altered the morphology of the nuclei only in the primary leukemic samples, increasing the number of micro/macronuclei and of DNA bridges. All the nuclear alterations were restricted to leukemic samples (Fig. 3d). Our findings in the primary samples are consistent with those observed in the cell lines, suggesting a consistent mechanism of cell death.

### AZD-1775 sensitizes leukemia cells to clofarabine and other genotoxic agents

**Fig. 4:**
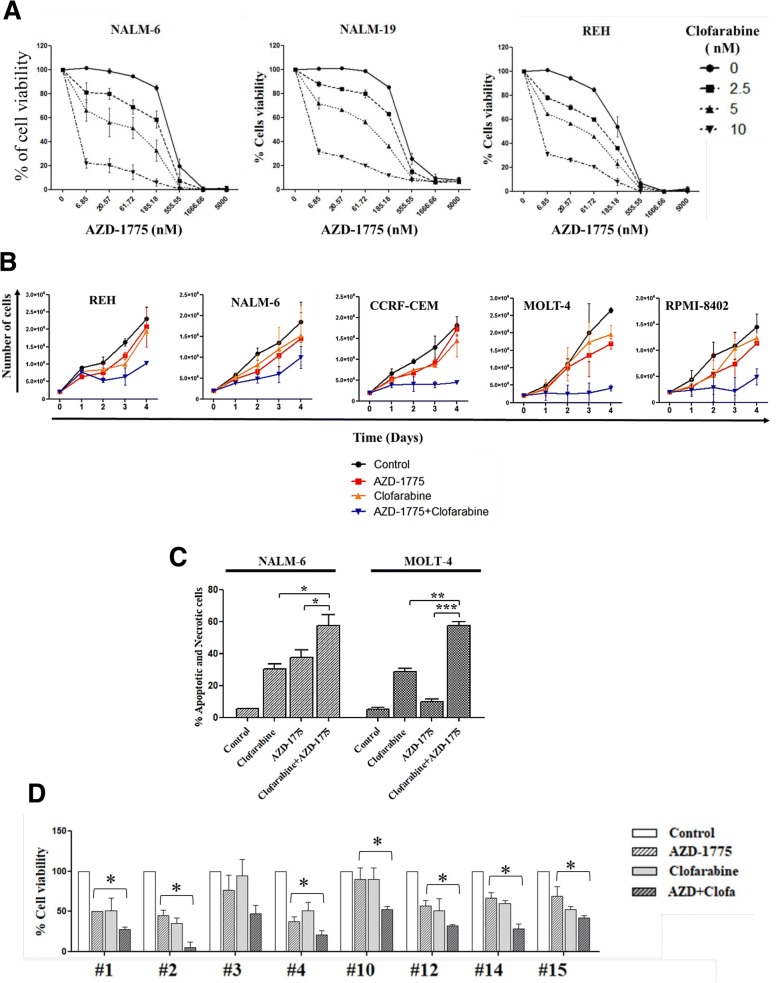
AZD-1775 enhances the toxicity of antineoplastic compounds on ALL cell lines and primary cells. **a** Cell viability analyses of NALM-6, NALM-19, and REH cell lines incubated with AZD-1775 (6 to 5000 nM, dilution rate 1:3) and clofarabine (2.5, 5, and 10 nM) for 72 h. Viable cells are depicted relative to the untreated controls. Data were used to determine the CI values. **b** Growth curve of REH, NALM-6, CCRF-CEM, MOLT-4, and RPMI-8402 treated for 4 days with AZD-1775 (185 nM) and clofarabine (REH, NALM-6, and RPMI-8402 10 nM; CCRF-CEM and MOLT-4 20 nM). The number of viable cells was evaluated in the different groups every 24 h. **c** Apoptosis analyses of NALM-6 and MOLT-4 cell lines after 24 h of incubation with AZD-1775 (185 nM) and clofarabine (MOLT-4 20 nM and NALM-6 10 nM). The percentage of apoptotic cells was detected after Annexin V/propidium iodide staining. **d** Cell viability analysis on primary leukemic cells isolated from eight adult ALL patients treated with AZD-1775 (5 uM) and clofarabine (500 nM) for 24 h. Viable cells are depicted as percentage of the untreated controls. *p ≤ 0.05, **p ≤ 0.01, ***p ≤ 0.001

The current therapeutic approaches for both *BCR*-*ABL1*-positive/negative ALL patients are based on conventional chemotherapy with or without targeted therapeutics (for example, tyrosine kinase inhibitors, TKIs), especially during the induction phase [3, 49]. The findings demonstrating that AZD-1775 can act to increase the efficacy of DNA damaging drugs, i.e., can act as a chemo-sensitizer agent [36, 37, 50], promoted us to evaluate whether AZD-1775 could also play a role as a chemo-sensitizer with drugs routinely used in clinical practice for ALL patients. Different ALL cell lines were treated for 24, 48, and 72 h with increasing concentration of AZD-1775 (from 6 to 5000 nM) in combination with increasing concentration of the antimetabolite clofarabine (NALM-6, NALM-19, REH: 2.5, 5, 10 nM, respectively; CCRF-CEM, MOLT-4, and RPMI-8402: 5, 10, 20 nM, respectively). Combination index (CI) analyses were performed on each cell line to determine any synergism, additivity, or antagonism of the different drug combinations. However, AZD-1775 generally acted with clofarabine in synergism (Fig. 4a; Table 1 for CI summary). Based on the results of the CI analyses, MOLT-4 and NALM-6 cell lines were treated with AZD-1775 (185 nM) in combination with clofarabine (MOLT-4 20 nM and NALM-6 10 nM), and cell proliferation was assessed. The combined treatment for 4 days induced a reduction in proliferation of B/T-ALL cell lines with the most dramatic results observed in MOLT-4, CCRF-CEM, and RPMI-8402 cell lines (Fig. 4b). Similar results on the reduction of proliferation were obtained with AZD-1775 in combination with the topoisomerase 2 inhibitor, doxorubicin, which also results in DNA damage (Additional file 2: Figure S2A). We next evaluated if the reduction of cell viability and proliferation was due to the induction of apoptosis. In both cell lines, the combination significantly increased the percentage of apoptotic cells, in comparison with the effect of clofarabine (MOLT-4, *p* = 0.0029; NALM-6, *p* = 0.0171) or AZD-1775 (MOLT-4, *p* = 0.0008; NALM-6, *p* = 0.0377) as single agents (Fig. 4c). We also confirmed the efficacy of the combined treatment on primary cells isolated from eight adult *BCR*-*ABL1*-negative ALL patients, with the exception of sample #3, in comparison with the effect of the single agent treatments (*p* < 0.05) (Fig. 4d).

**Table 1:** Combination index values of AZD-1775 in combination with clofarabine

			24 h			48 h			72 h		
			Clofarabine (nM)			Clofarabine (nM)			Clofarabine (nM)		
Cell lines			2.5	5	10	2.5	5	10	2.5	5	10
REH	AZD-1775 (nM)	6.9	0.70	0.80	0.95	0.61	0.88	0.99	0.78	1.07	1.12
		20.6	0.80	0.70	0.94	0.85	0.95	1.06	0.78	1.02	1.15
		61.7	1.32	0.70	0.91	1.61	1.38	1.05	0.90	1.10	1.09
		185.2	1.64	0.70	0.82	2.56	2.02	0.92	1.26	1.09	0.81
		555.6	0.89	0.46	0.65	1.00	0.82	0.55	0.43	0.24	0.29
		1666.7	1.10	0.43	0.76	0.33	0.34	0.34	0.62	0.64	0.68
		5000.0	2.23	1.11	0.77	1.00	0.26	1.01	1.81	1.83	1.88
NALM-6	AZD-1775 (nM)	6.9	0.24	0.45	0.55	0.60	0.46	0.75	0.73	1.06	1.15
		20.6	0.20	0.30	0.49	0.49	0.56	0.96	0.76	1.02	1.17
		61.7	0.26	0.22	0.39	0.66	0.76	0.93	0.84	1.08	1.14
		185.2	0.45	0.34	0.33	1.99	1.31	0.80	1.24	1.16	0.97
		555.6	0.63	0.39	0.27	1.38	0.93	0.66	0.85	0.66	0.57
		1666.7	0.55	0.36	0.33	9.87	0.84	1.04	0.20	0.26	0.37
		5000.0	0.84	0.64	0.71	2.05	1.98	2.19	0.49	0.55	0.66
NALM-19	AZD-1775 (nM)	6.9	0.46	0.54	0.85	0.52	0.59	0.81	1.02	1.20	1.09
		20.6	0.45	0.43	0.60	0.35	0.54	0.68	1.00	1.18	1.08
		61.7	0.56	0.39	0.62	0.38	0.41	0.43	0.91	1.01	0.92
		185.2	0.73	0.32	0.44	0.49	0.35	0.20	0.91	0.79	0.73
		555.6	0.60	0.21	0.32	0.20	0.14	0.18	0.53	0.50	0.74
		1666.7	0.68	0.27	0.41	0.21	0.15	0.27	0.69	0.75	1.10
		5000.0	1.39	0.76	0.69	0.56	0.53	0.70	1.75	1.79	2.43
RPMI-8402	AZD-1775 (nM)	6.9	0.21	0.26	0.50	0.23	0.36	1.39	8.20	1.23	1.46
		20.6	0.14	0.24	0.63	0.34	0.42	11.91	0.69	0.78	1.43
		61.7	0.22	0.21	0.62	0.77	0.53	3.17	0.55	0.83	1.26
		185.2	0.41	0.26	0.81	1.48	1.43	2.37	0.66	0.80	0.73
		555.6	0.57	0.36	0.54	0.95	0.61	0.48	0.43	0.41	0.08
		1666.7	0.60	0.38	0.32	0.55	0.33	0.21	0.28	0.10	0.12
		5000.0	0.37	0.26	0.05	0.09	0.02	0.04	0.22	0.24	0.26
Cell lines			5	10	20	5	10	20	5	10	20
CCRF-CEM	AZD-1775 (nM)	6.9	0.01	0.02	0.23	0.74	1.13	1.15	1.01	1.61	1.06
		20.6	0.01	0.01	0.08	0.92	2.29	1.30	0.43	2.41	1.02
		61.7	0.01	0.01	0.02	0.64	1.07	0.70	4.20	4.81	0.98
		185.2	0.01	0.01	0.02	1.13	0.78	0.29	0.91	0.83	0.76
		555.6	0.01	0.01	0.01	0.20	0.19	0.22	0.39	0.51	0.72
		1666.7	0.05	0.06	0.09	0.44	0.42	0.45	0.80	0.94	1.08
		5000.0	0.42	0.45	0.30	1.40	1.22	0.96	2.02	2.11	1.87
MOLT-4	AZD-1775 (nM)	6.9	0.00	0.09	0.25	1.62	8.99	8.15	1.00	1.03	1.27
		20.6	0.11	0.15	0.32	2.26	0.85	10.07	0.80	1.14	1.02
		61.7	0.32	0.24	0.40	0.69	0.75	0.55	0.95	1.19	0.62
		185.2	0.57	0.27	0.23	1.24	0.94	0.43	0.81	0.53	0.26
		555.6	0.31	0.16	0.18	0.88	0.61	0.40	0.00	0.11	0.12
		1666.7	0.40	0.28	0.30	1.31	1.42	0.42	0.31	0.31	0.32
		5000.0	1.61	1.76	1.66	6.06	5.49	5.24	0.92	2.04	0.93

The table reports the Combination Index (CI) value for each combination of drugs. CI < 1 indicates synergism, CI = 1 indicates additivity and CI > 1 indicates antagonism

### AZD-1775 sensitizes leukemic cells to the tyrosine kinase inhibitors

**Fig. 5:**
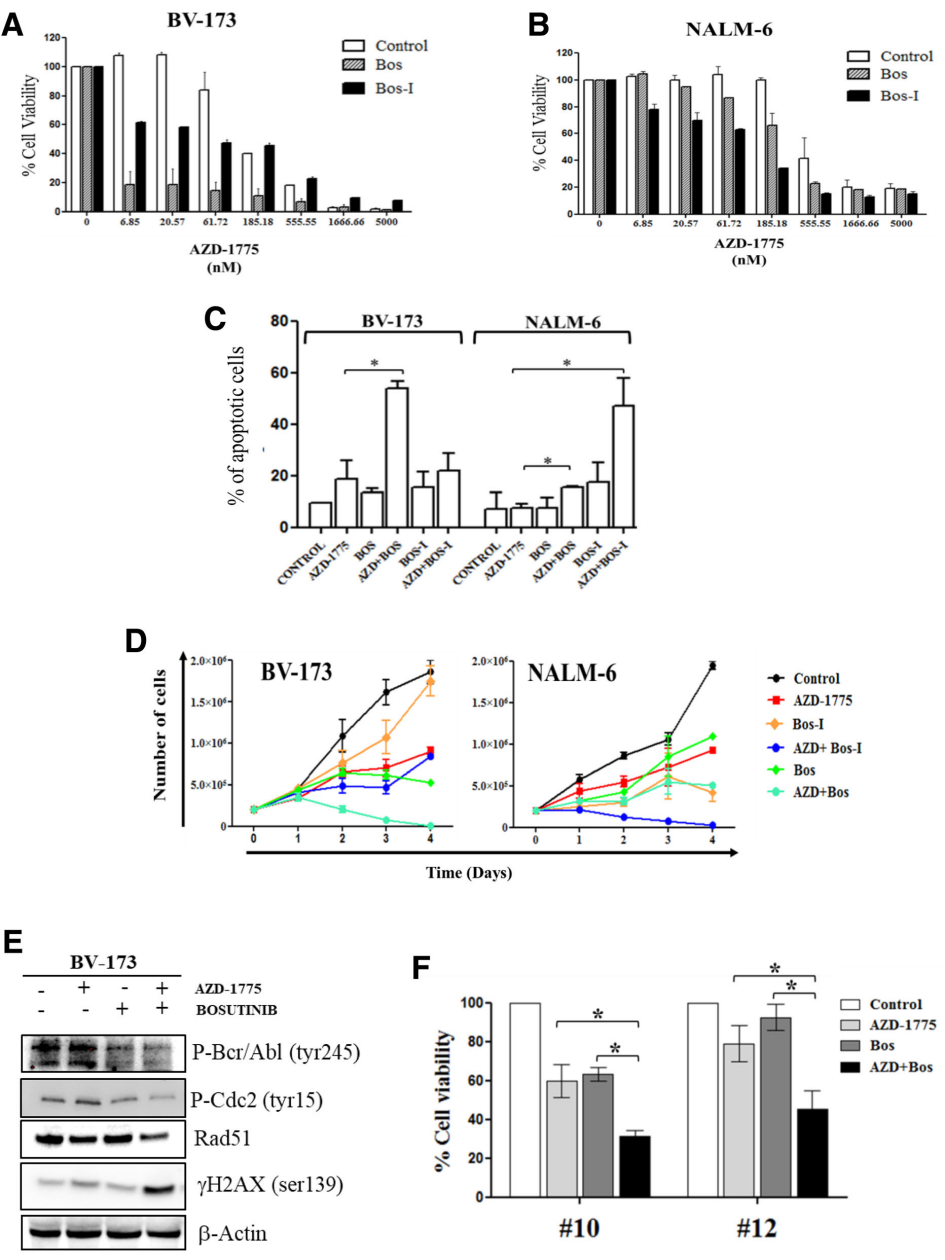
AZD-1775 enhances the toxicity of tyrosine kinase inhibitors in ALL cell lines and leukemia primary samples. **a** Cell viability analyses of BV-173 cell line incubated simultaneously with AZD-1775 (6 to 5000 nM, dilution rate 1:3) and with bosutinib authentic (50 nM) or bosutinib isomer (50 nM) for 48 h. In the graph: AZD-1775 (control, white columns), bosutinib (Bos, gray columns), and bosutinib isomer (Bos-I, black columns). The percentage of viable cells is depicted relative to the untreated controls. Data were used to determine CI values. **b** Cell viability analyses of NALM-6 cell line incubated with AZD-1775 (6 to 5000 nM, dilution rate 1:3) and with bosutinib authentic (2 uM) or bosutinib isomer (2 uM) for 72 h. In the graph: AZD-1775(control, white columns), bosutinib (Bos, gray columns), and bosutinib isomer (Bos-I, black columns). The number of viable cells is depicted as a percentage of the untreated controls. Data were used to determine CI values. **c** Apoptosis analyses of BV-173 and NALM-6 cells after 24 h of incubation with AZD-1775 (185 nM) and bosutinib authentic/isomer (BV-173 50 nM; NALM-6 2 uM). The percentage of apoptotic cells was detected after Annexin V/propidium iodide staining. **d** Growth curve of BV-173 and NALM-6 treated for 4 days with AZD-1775 (185 nM) and bosutinib authentic/bosutinib isomer (BV-173 50 nM; NALM-6 2 uM). **e** Immunoblotting analysis of BV-173 treated with AZD-1775 (185 nM) and bosutinib (50 nM) for 24 h. β-actin was used for loading normalization. **f** Cell viability analysis in primary leukemic cells isolated from two adult *BCR*-*ABL1*-positive ALL patients treated with AZD-1775 (5 uM) and bosutinib authentic (2 uM) for 24 h. The percentage of viable cells is depicted relative to the untreated controls. Controls in all panels are cells treated with DMSO 0.1%. *p ≤ 0.05

The chemo-sensitizing activity of AZD-1775 was also assessed in combination with different TKIs, which represent the frontline therapy for the treatment of *BCR*-*ABL1*-positive ALL and chronic myeloid leukemia (CML) patients [51–54]. Specifically, we explored the effect of AZD-1775 in combination with bosutinib (Bos), which is approved by the FDA for newly diagnosed *BCR*-*ABL1*-positive CML. In addition, we evaluated a structural isomer of bosutinib (hence named as Bos-I). We decided to combine TKIs with AZD-1775 as we previously showed that both Bos and Bos-I had off-target inhibitory activity on WEE1 and CHK1 kinases (albeit greater inhibitory activity is observed for Bos-I versus Bos) [28, 55] and also that this compound can sensitize pancreatic tumor cells to the anti-metabolite gemcitabine [28]. The efficacy of the two isomers as a single agent was performed on the panel of ALL cell lines. Both compounds reduced the viability of ALL cell lines, although Bos-I resulted in more potent anti-proliferative activity than Bos on *BCR*-*ABL1*-negative cell lines (Additional file 2: Figure S2B). The combination of Bos (subtoxic concentration) with AZD-1775 using increasing concentration (from 6 to 5000 nM, 1:3 dilution series) for 24 and 48 h reduced the cell viability not only of *BCR*-*ABL1*-positive cell lines but also of different *BCR*-*ABL1*-negative cell lines. Interestingly, this combination had a stronger effect on *BCR*-*ABL1*-positive cell lines than on the combination between AZD-1775 and Bos-I (Fig. 5a). However, in *BCR*-*ABL1*-negative cell lines, the combination of AZD-1775 with Bos-I had a greater effect on reducing cell viability in comparison with Bos (Fig. 5b; Tables 2 and 3 for CI summary). To investigate the effect of the drugs on apoptosis induction and proliferation, BV-173 and NALM-6 were treated for 24 h with AZD-1775 (185 nM) and sublethal concentrations of Bos or Bos-I (BV-173 50 nM and NALM-6 2uM). The comparison of the two combinations showed that AZD-1775 combined with Bos significantly increased the percentage of apoptotic cells in *BCR*-*ABL1*-positive BV-173 cells. In contrast, AZD-1775 combined with Bos-I was more effective in inducing apoptosis of *BCR*-*ABL1*-negative NALM-6 cells (Fig. 5c). Similar results were observed on the inhibition of cell proliferation (Fig. 5d). To expand upon the data of the synergism between AZD-1775 and TKIs on *BCR*-*ABL1*-positive cells, we tested other TKIs indicated for CML and ALL. Different combination index analyses using increasing concentration of AZD-1775 (from 6 to 5000 nM; dilution 1:3) in combination with ponatinib (25, 50, and 100 nM) or imatinib (250, 500, and 1000 nM) were performed on BV-173 for 24 and 48 h and showed comparable results in terms of synergy (Additional file 2: Figure S2D; Table 4 for CI summary). To explain the biological reasons for the synergic effect between AZD-1775 and the TKIs, immunoblotting analysis was performed on BV-173 cell line. The combination treatment of AZD-1775 (185 nM) and bosutinib (50 nM) enhanced γH2AX, the marker of DNA damage. The combination also perturbed the G2/M checkpoint as evidenced by the combined effect on reducing phospho-CDC2 (Tyr15) in comparison with the single treatments. Different studies showed that the oncogenic BCR/ABL tyrosine kinase facilitates the repair of DNA double-strand breaks (DSBs) through the stimulation of the homologous recombination (HR) repair [56]. It also has been established that AZD-1775 impairs HR repair through forced activation of CDC2 [57]. Based on this knowledge, the synergism of the combination could be related to the impairment of the DNA repair machinery. Consistent with this notion, the combination treatment additively reduced the amount of RAD51 protein master regulator of DSB repair to the HR repair [58]. In addition, the putative effect of AZD-1775 on altering BCR/ABL1 functionality was evaluated by looking at the expression of phospho-BCR/ABL (tyr245) fusion protein. Indeed, no effect of AZD-1775 was seen on BCR/ABL1 levels (Fig. 5e). Finally, we observed that AZD-1775 sensitized also the primary cells isolated from two BCR-ABL1-positive ALL patients to the TKIs (Fig. 5f), in concordance with our data in cell lines, suggesting a consistent mechanism of action.

**Table 2:** Combination index values of AZD-1775 in combination with bosutinib (Bos) on *BCR-ABL1*-positive cell lines

			24 h			48 h		
			BOS (nM)			BOS (nM)		
Cell line			10	25	50	10	25	50
BV-173	AZD-1775 (nM)	6.9	0.95	1.11	0.99	1.45	1.03	1.27
		20.6	1.03	1.21	1.01	1.59	0.98	1.29
		61.7	0.73	0.86	0.62	0.88	0.72	1.08
		185.2	0.71	0.77	0.71	0.59	0.65	1.01
		555.6	1.26	0.75	0.61	0.85	0.78	1.03
		1666.7	1.14	0.92	0.81	1.08	1.08	1.24
		5000.0	1.82	1.71	1.58	2.44	1.86	2.02
Cell line			500	1000	2000	500	1000	2000
SUP-B15	AZD-1775 (nM)	6.9	0.52	0.96	1.23	0.59	0.84	1.23
		20.6	0.64	0.72	1.11	0.61	0.99	0.98
		61.7	0.7	1.36	0.91	0.73	0.83	0.89
		185.2	1.05	1.51	0.79	0.79	0.88	0.79
		555.6	1.07	1.3	1.14	1.18	1.22	1
		1666.7	0.44	0.54	0.46	0.5	0.55	0.54
		5000.0	0.51	0.49	0.4	0.41	0.41	0.31

The table reports the Combination Index (CI) value for each combination of drugs. CI < 1 indicates synergism, CI = 1 indicates additivity and CI > 1 indicates antagonism

**Table 3:** Combination index values of AZD-1775 in combination with bosutinib isomer (Bos-I) on *BCR-ABL1*-negative cell lines

			24 h			48 h			72 h		
			Bosutinib isomer (nM)			Bosutinib isomer (nM)			Bosutinib isomer (nM)		
Cell line			500	1000	2000	500	1000	2000	500	1000	2000
NALM-6	AZD-1775 (nM)	6.9	0.57	0.69	0.79	0.62	1.00	1.21	0.59	0.79	1.14
		20.6	0.33	0.48	0.64	0.65	0.81	1.07	0.47	0.68	0.99
		61.7	0.35	0.39	0.65	0.67	0.65	0.97	0.68	0.73	0.93
		185.2	0.48	0.41	0.41	0.91	0.65	0.68	0.89	0.73	0.63
		555.6	0.63	0.43	0.36	0.54	0.45	0.47	0.41	0.40	0.48
		1666.7	0.67	0.51	0.41	0.65	0.66	0.68	0.53	0.59	0.66
		5000.0	1.65	1.43	1.21	1.58	1.61	1.62	1.42	1.43	1.52
Cell lines			250	500	1000	250	500	1000	250	500	1000
NALM-19	AZD-1775 (nM)	6.9	0.43	0.75	1.01	1.99	2.94	0.20	0.19	0.28	0.35
		20.6	0.38	0.47	0.63	1.53	1.15	0.12	0.24	0.29	0.28
		61.7	0.19	0.27	0.23	0.20	0.12	0.09	0.44	0.48	0.31
		185.2	0.27	0.24	0.24	0.42	0.23	0.15	0.67	0.48	0.26
		555.6	0.38	0.31	0.28	0.41	0.31	0.25	0.29	0.28	0.21
		1666.7	0.76	0.68	0.69	0.82	0.72	0.69	0.47	0.44	0.38
		5000.0	1.78	1.95	1.77	2.04	2.09	1.93	1.19	1.30	1.13
MOLT-4	AZD-1775 (nM)	6.9	1.23	0.93	1.11	0.99	1.03	1.28	0.67	1.22	1.26
		20.6	0.78	0.61	0.78	0.85	0.89	1.07	0.74	0.89	1.07
		61.7	0.77	0.53	0.43	0.62	0.63	0.71	0.91	0.85	0.77
		185.2	0.53	0.40	0.23	0.39	0.35	0.34	0.35	0.37	0.53
		555.6	0.22	0.25	0.21	0.34	0.40	0.50	0.35	0.43	0.67
		1666.7	0.40	0.60	0.65	0.95	1.12	1.14	0.92	0.96	1.17
		5000.0	2.46	2.25	3.02	3.06	3.26	3.49	3.54	3.30	3.52
Cell line			2000	5000	10000	2000	5000	10000	2000	5000	10000
CCRF-CEM	AZD-1775 (nM)	6.9	0.75	0.78	0.84	0.75	0.78	0.84	1.06	0.65	0.39
		20.6	0.38	0.62	0.79	0.38	0.62	0.79	1.21	0.57	0.52
		61.7	0.27	0.54	0.74	0.27	0.54	0.74	0.53	0.49	0.52
		185.2	0.26	0.46	0.58	0.26	0.46	0.58	0.28	0.43	0.49
		555.6	0.25	0.65	0.53	0.25	0.65	0.53	0.35	0.49	0.37
		1666.7	0.84	0.93	0.81	0.84	0.93	0.81	0.66	0.79	0.46
		5000.0	3.13	2.76	0.99	3.13	2.76	0.99	2.08	1.97	0.88

The table reports the Combination Index (CI) value for each combination of drugs. CI < 1 indicates synergism, CI = 1 indicates additivity and CI > 1 indicates antagonism

**Table 4:** Combination index values of AZD-1775 in combination with ponatinib and imatinib on BV-173 cell lines

			24 h			48 h		
			Ponatinb (nM)			Ponatinb (nM)		
Cell line			25	50	100	25	50	100
BV-173	AZD-1775 (nM)	6.9	0.39	0.57	1.16	0.29	0.31	0.67
		20.6	0.34	0.59	1.04	0.33	0.34	0.50
		61.7	0.33	0.55	0.99	0.16	0.17	0.56
		185.2	0.31	0.49	0.90	0.06	0.11	0.48
		555.6	0.47	0.53	0.83	0.02	0.07	0.20
		1666.7	0.53	0.58	0.86	0.01	0.01	0.12
		5000.0	1.23	1.33	1.38	0.06	0.07	0.15
			Imatinib (nM)			Imatinib (nM)		
Cell line			250	500	1000	250	500	1000
BV-173	AZD-1775 (nM)	6.9	0.39	0.41	0.62	0.41	0.03	0.02
		20.6	0.58	0.43	0.69	0.43	0.04	0.03
		61.7	0.32	0.33	0.39	0.18	0.06	0.05
		185.2	0.23	0.31	0.47	0.15	0.07	0.05
		555.6	0.50	0.44	0.48	0.11	0.08	0.07
		1666.7	0.57	0.50	0.53	0.07	0.20	0.17
		5000.0	1.05	1.18	1.21	0.61	1.19	1.56

The table reports the Combination Index (CI) value for each combination of drugs. CI < 1 indicates synergism, CI = 1 indicates additivity and CI > 1 indicates antagonism

## Discussion

The inhibition of the DDR is a promising therapeutic strategy to sensitize tumor cells to an additional therapeutic compound, with the goal to improve response rates for the treatment of various cancers. Indeed, this rational approach is being thoroughly investigated in clinical studies with over 15 trials currently evaluating the efficacy of a DDR-inhibitor in combination with DNA damaging agents. For example, AZD-1775 is being evaluated in combination with cisplatin for breast cancer (NCT03012477) or with carboplatin and paclitaxel for squamous cell lung cancer (NCT02513563). Nonetheless, no trial has been yet registered to assess this approach to treat ALL. Our group has previously reported the efficacy of different CHK1/CHK2 inhibitors as a single agent and in combinations in primary ALL samples [43], suggesting that this approach might be amenable for the treatment of ALL in a clinical setting. Following this line of thought, we asked whether other DDR targets in ALL may also be efficaciously inhibited. Here, we focused on WEE1, since we observed that *WEE1* is highly expressed in primary leukemic samples from adult ALL patients and, in particular, in the relapsed samples, confirming previous data in solid tumors from multiple studies [24, 27, 59–62]. Moreover, the significance of *Wee1* expression in ALL is further exemplified by the data from the CCLE database [63] showing that out of twenty profiled indications, B-ALL and T-ALL have the fourth and fifth highest expression of *Wee1* mRNA, respectively (Additional file 2: Figure S3A). Regarding the correlation between Wee1 mRNA and protein expression, in 5/7 cases, our results provide concordant data and suggest that a 50% cutoff of WEE1-positive blasts may reliably indicate upregulated Wee1 mRNA; in addition, the WEE1 immunohistochemical negativity always corresponds to Wee1 mRNA downregulation. It is tempting to speculate that these data, together with our previous findings [43, 44], suggest that in primary leukemic cells, the expression of several DDR genes may be fundamental to sustain the genetic instability, to overcome the inhibitory signal of DNA damage checkpoint activation, and to promote proliferation [26]. Indeed, the bypass of the DNA damage checkpoint activation by pharmacological inhibition of WEE1 led to the increased DNA damage (as measured by γ-H2AX), drastically reduced cell viability, inhibited the proliferation rate, and induced apoptosis both in ALL cell lines and primary leukemic blasts. Considering the effect of AZD-1775 on perturbation of the cell cycle (increase of cells in S and G2/M phases) and on cell death, we hypothesize that the induction of cell death must occur during late S phase or mitosis. Indeed, in the primary leukemic samples, we observed the appearance of DNA bridges, which are markers of aberrant mitosis, and we found that the apoptotic bodies were strongly positive for the phospho-MPM2 marker.

It has been established that, in order to prevent premature entry in mitosis, CDK1 is maintained in an inactive state by WEE1 through phosphorylation on tyrosine 15 and, subsequently, by MYT1 (PKMYT1) through phosphorylation on threonine 14 [64]. The importance of MYT1 has not yet been fully understood, especially in cancer cells. Here, we report that in our ALL cohort, *MYT1* gene is highly expressed in relapsed *BCR*-*ABL1*-positive/negative ALL samples in comparison with normal MNCs (Additional file 2: Figure S1F). Moreover, in the ex vivo drug treatment, the samples highly sensitive to AZD-1775 showed a higher level of expression of *MYT1* (*p* = 0.004) in comparison with the intermediate, poor, and MNCs groups. While the significance of this finding is beyond the scope of this study, it does hint at MYT1 playing a role in ALL. In agreement with the results by Tibes and colleagues [61], our data demonstrated that WEE1 inhibition leads to chemo-sensitization in ALL cell lines and in primary leukemic samples, albeit using a different approach. In that study, an unbiased RNAi screen looking for sensitizers to cytarabine identified WEE1 as the top hit, and the authors further validated these results using AZD-1775. Additionally, they found that sensitization occurred in AML and CML cell lines, suggesting the use of these rational drug combinations with WEE1 inhibitor in other hematological indications.

In *BCR*-*ABL1*-positive ALL samples, the concomitant inhibition of WEE1 and *BCR*-*ABL1* resulted in a significant inhibition of cell viability and proliferation as well as induction of apoptosis. Consistently, taking advantage of the different kinase inhibitory profiles of Bos and Bos-I toward WEE1 [28], Bos-I results in approximately tenfold more potent synergistic activity. It is interesting to note that Bos-I displayed greater synergistic activity in *BCR*-*ABL1*-negative (not Ph-like) cell lines than Bos. We hypothesize that the observed synergy derived from the combined treatment of AZD-1775 with the two TKI compounds is not mediated through their on-target activity toward Src and Abl as this is similar between the two compounds [28]. Indeed, this notion is consistent with the finding that AZD-1775 synergizes with imatinib and ponatinib. One possible explanation of the synergism between AZD-1775 and TKIs comes from the effect of BCR-ABL fusion protein on G2/M checkpoint and DNA repair in leukemic cells [56, 65–67]. In this scenario, the synergism of the combination may be due to the effect of the two classes of inhibitors on the G2/M checkpoint stability and on the functionality of the DNA repair pathway.

## Conclusions

Our findings suggest that WEE1 may play a role in the leukemogenesis and in the proliferation of ALL blasts. The inhibition of WEE1, in monotherapy or in combination with different antineoplastic agents, results in a significant reduction of cell viability and apoptosis induction. Although additional in vivo studies should be performed to enforce our results, it has been shown that in ALL models, there is a good correlation of drug studies using ex vivo ALL primary cells and then testing them in vivo model (PDX) [68]. These data lay the basis for evaluation of AZD-1775 as a chemo-sensitizer in the clinic for the treatment of ALL.

## Additional files

Additional file 1: **Table S1.** Patient’s characteristic of gene expression cohort. Table S2. Patient’s characteristic ex vivo AZD-1775 treatment in single agent or in combination. Table S3. Quantitative analyses of G2/M checkpoint-related genes. Differential gene expression of 24 genes involved in the regulation of the G2/M checkpoint of primary leukemic cells in comparison to normal mononuclear cells (MNCs). In the table, the primary leukemic samples have been divided into three groups based on the ex vivo sensitivity to AZD-1775. Very good IC_50_ < 5uM; good IC_50_ < 10uM; poor IC_50_ > 10 uM. (PDF 253 kb)

Additional file 2: **Figure S1.** Efficacy of AZD-1775 used as single agent. A) The graph shows the IC_50_ values of B/T-ALL cell lines treated with AZD-1775 for 24, 48, and 72 h. B) Cell viability analysis on CCRF-CEM cell lines showing the effect of high doses of AZD-1775. The percentage of viable cells is depicted relative to untreated controls. C) Immunoblot analysis on BV-173 treated with AZD-1775 (IC_50_) for 12 h. D) Cell cycle analysis in BV-173 and CCRF-CEM cell lines treated with AZD-1775 (IC_50_) for 12 h. E) Immunofluorescence analysis of BV-173 cells treated with AZD-1775 (IC_50_) for 12 h and, then, stained with DAPI and phospho-MPM2. In the picture, a cell dying in mitosis is reported with apoptotic bodies strongly positive for phospho-MPM2 antibody. Representative images are shown at × 100 magnification. F) Viability of mononuclear cells isolated from the peripheral blood of 5 healthy donors incubated with increasing concentration of AZD-1775 (2.5, 5, and 10 uM) for 24 h. G) MYT1 transcript levels in samples isolated from adult *BCR*-*ABL1*-positive ALL at diagnosis (*n* = 17), adult *BCR*-*ABL1*-negative ALL at diagnosis (*n* = 27), adult *BCR*-*ABL1*-positive ALL at relapse (unpaired, *n* = 8), adult *BCR*-*ABL1*-negative ALL at relapse (unpaired, *n* = 6), and in MNCs (*n* = 7) from the peripheral blood of healthy donors. One-way ANOVA test was performed to assess statistical significance. Results are expressed as Log10 2 exp.[−(ΔΔCt). **Figure S2.** AZD-1775 in combination with chemotherapy agents and tyrosine kinase inhibitors. A) Growth curve of BV-173 and REH cell lines treated for 4 days with AZD-1775 (185 nM) and doxorubicin (25 nM). B) Viability analyses in ALL cell lines incubated for 24 h with Bos or Bos-I (6 to 5000 nM). The percentage of viable cells is depicted relative to untreated controls. C) Cell viability analysis of BV-173 cell line treated with AZD-1775 (6 to 5000 nM, dilution rate 1:3) and with ponatinib (25, 50, 100 nM) or imatinib (250, 500, and 1000 nM) for 24 h. The percentage of viable cells is depicted relative to untreated controls, 0.1%. ** Figure S3**. *Wee1* mRNA expression across different cancer types from the Cancer Cell Line Encyclopedia (CCLE) database. A) Box plots showing the level of expression of *Wee1* mRNA in different tumor samples, extracted from CCLE [63]. The red arrows point to B/T-ALL samples. Boxes define the 25th and the 75th percentiles, horizontal line within the boxes indicates the median, and whiskers define the 10th and the 90th percentiles. (PDF 1918 kb)

## Abbreviations

ALL: Acute lymphoblastic leukemia
BOS: Bosutinib
BOS-I: Bosutinib isomer
CCNB1/B2: Cyclin B1/B2
CDC2: Cell division cycle protein 2 homolog
CDC25: Cell division cycle 25
CHK1: Checkpoint kinase 1
CHK2: Checkpoint kinase 2
DDR: DNA damage response
MNC: Mononuclear cell
MPF: Mitotic promoting factor
MYT1 (PK MYT1): Membrane-associated tyrosine/threonine 1
TKI: Tyrosine kinase inhibitor
