# Supplementary material for: Targeting WEE1 to enhance conventional therapies for acute lymphoblastic leukemia
Source: J Hematol Oncol. 2018 Aug 1;11:99. doi: 10.1186/s13045-018-0641-1 (PMC6090987; doi:10.1186/s13045-018-0641-1)
Supplement: Supplementary file 1 — Table S1. Patient’s characteristic of gene expression cohort. Table S2. Patient’s characteristic ex vivo AZD-1775 treatment in single agent or in combination. Table S3. Quantitative analyses of G2/M checkpoint-related genes. Differential gene expression of 24 genes involved in the regulation of the G2/M checkpoint of primary leukemic cells in comparison to normal mononuclear cells (MNCs). In the table, the primary leukemic samples have been divided into three groups based on the ex vivo sensitivity to AZD-1775. Very good IC50 < 5uM; good IC50 < 10uM; poor IC50 > 10 uM. (PDF 253 kb) [file 13045_2018_641_MOESM1_ESM.pdf]

Table S1. Patient's characteristic of gene expression cohort

SAMPLE ID	**BCR-ABL1 translocation**	**Stage of the disease**	**Karyotype**	**Type of samples**	**WEE1 relative transcript level**	**MYT1 relative transcript level**
ALL_1	NEGATIVE	DIAGNOSIS	NA	PBL	11.40067	5.113971
ALL_2	POSITIVE	RELAPSE	NA	PBL	9.41669	4.941288
ALL_3	NEGATIVE	DIAGNOSIS	NA	BM	12.86321	5.010772
ALL_4	NEGATIVE	DIAGNOSIS	NA	BM	7.901237	5.247456
ALL_5	NEGATIVE	DIAGNOSIS	NA	BM	10.46552	4.703445
ALL_6	POSITIVE	DIAGNOSIS	46,XX [10]	BM	9.873922	5.619489
ALL_7	POSITIVE	DIAGNOSIS	46XY,t(9;22)(q34;q11)[1]/46XY[2]	BM	8.034962	4.735756
ALL_8	NEGATIVE	DIAGNOSIS	47,XX,+8 [16]/46,XX [4]	BM	9.538575	5.238142
ALL_9	POSITIVE	DIAGNOSIS	46XX, t(9;22)(q34;q11)[1]. 47XX, +der22 t(9;22)[1].46XX, -7, der12 t(7;12)(q11.2,p13),+der22, t(9;22)[5]	PBL	7.667279	4.832946
ALL_10	NEGATIVE	DIAGNOSIS	NA	PBL	8.896559	4.913634
ALL_11	POSITIVE	DIAGNOSIS	NA	PBL	8.259012	4.687167
ALL_12	POSITIVE	DIAGNOSIS	NA	BM	9.566517	5.41777
ALL_13	NEGATIVE	DIAGNOSIS	46 XY [20] (t(12;21) neg)	PBL	10.58061	5.574841
ALL_14	POSITIVE	DIAGNOSIS	46,XY [28]	BM	8.941937	5.278478
ALL_15	NEGATIVE	DIAGNOSIS	46 XX	BM	9.126612	5.552184
ALL_16	POSITIVE	DIAGNOSIS	45XX, t(9;22)(q34;q11), der(16)dic(16;20)(q13;11), -20 [20]	BM	8.994265	4.775842
ALL_17	NEGATIVE	RELAPSE	45XY,-7[9]/45XY,-7,del (9)[3]/46XY [12] /// [DX: 46XY [20]/81chrs [1] nv]	BM	9.416733	5.210949
ALL_18	NEGATIVE	DIAGNOSIS	46XY [9]	BM	9.894938	4.975881
ALL_19	NEGATIVE	DIAGNOSIS	46 XX	BM	10.72273	5.329332
ALL_20	NEGATIVE	DIAGNOSIS	NA	PBL	9.416162	4.67443
ALL_21	POSITIVE	DIAGNOSIS	46,XY, t(9;22)(q34;q11)	BM	8.839748	5.196205
ALL_22	NEGATIVE	DIAGNOSIS	46XX,der(6)t(1;6)(q11;q11)t(8;14)(q24;q32)del9q21,add(13)(q34)[16/16]	BM	11.14958	5.47596
ALL_23	NEGATIVE	DIAGNOSIS	46XY [11]/68-71,XXY,-X,-X,-Y,+1,+5,-7,-9,-10,+11,-13,-18,+2 [9]	BM	10.722	4.961411
ALL_24	POSITIVE	DIAGNOSIS	46XX [13]/45 XX t(9;22)(q34;q11),-13,del(16)(q22) [7/20]	PBL	11.359	4.810295

ALL_25	POSITIVE	RELAPSE	45,XX,-7,t(9;22)(q34;q11)[1]/46,XX,-7,t(9;22)(q34;q11),+der(22)t(9;22)(q34;q11)[1]/46,XX,-7,der(9)t(1;9)(q24;q34),t(9;22)(q34;q11),+der(22)t(9;22)(q34;q11)[10]/46,XX,-7,t(1;13)(p36;q14),der(9)t(1;9)(q24;q34),t(9;22)(q34;q11),+der(22)t(9;22)(q34;q11)[2]	BM	11.206	5.943592
ALL_26	NEGATIVE	DIAGNOSIS	46XX, del (9) (13p22p)[12]/46XX[8]	BM	9.044448	5.826427
ALL_27	NEGATIVE	RELAPSE	NA	BM	9.867239	5.912407
ALL_28	POSITIVE	RELAPSE	47 XX,+5, t(9;22)(q34;q11)[12];47,XX,+5,t(9;22)(q11;q34), DEL(12)(p12)[4].48XX,+5,t(9;22)(q11;q34),+mar [2].	BM	8.220219	4.976336
ALL_29	NEGATIVE	DIAGNOSIS	46,XY [17]/ 46,XY,t(2;16)(q13;q22), t(11;14)(q23;q32) [3]	BM	10.92681	5.628103
ALL_30	POSITIVE	RELAPSE	45 XY del7, t(9;22)(q34;q11), der12 t(7;12)(q11;p11) [16/20], 46 XY [4/20]	BM		
ALL_31	POSITIVE	DIAGNOSIS	46XX,t(9;22) [20/20]	PBL	9.320931	4.92153
ALL_32	POSITIVE	DIAGNOSIS	45XY, -3,der(9)t(3:9)(q10;p10),t(9;22)(q34;q11)[16]/46XY [3]	BM	8.193635	4.984048
ALL_33	POSITIVE	RELAPSE	46XY [3];45XY,der(14)t(7;14)?,t(9;22)(q34;q11),der(16)t(1;16)?,-17 [2]	BM	8.371907	4.768384
ALL_34	NEGATIVE	DIAGNOSIS	46XX[6]/46 XX,t(12;17)(p13,q21)[3]	PBL	7.650279	4.793464
ALL_35	NEGATIVE	DIAGNOSIS	47,XY,del(3)(q13),+6,t(11;19)(q23;p13) [4]/46,XY [6]	PBL	11.13743	5.28073
ALL_36	POSITIVE	DIAGNOSIS	NA	PBL	7.480791	4.621633
ALL_37	NEGATIVE	RELAPSE	46,XY[20]	BM	9.457473	5.499914
ALL_38	POSITIVE	DIAGNOSIS	46XX[12], 46XXt(9;22)(q34;q11) [13],46XX del(9)(p13;p22) t(9;22)(q34;q11) [5],46 XX del(9)(p13;p22) t(9;22)(q34;q11)[1]	BM	10.71223	5.086938
ALL_39	POSITIVE	DIAGNOSIS	46XX, t(9;22) [19/20]	BM	9.410629	4.68372
ALL_40	NEGATIVE	DIAGNOSIS	46 XY [20]	BM	9.313448	4.959898
ALL_41	NEGATIVE	RELAPSE	NA	BM	9.362439	5.37394

ALL_42	POSITIVE	RELAPSE	47XY,der(8;9)(q10,q10),t(9;22)(q34,q11),t(8;9)(q10,q10)x2[24]/46XY[1];25/03/2014: 47,XY,der(8)dic(8;9)(p11;p11)t(9;22)(q34;q11),+der(8)dic(8;9)(p11;p11)t(9;22)(q34;q11),-9,t(9;22)(q34;q11),+der(22)t(9;22)(q34;q11)[17/20]	BM	10.04857	5.356109
ALL_43	NEGATIVE	DIAGNOSIS	46XY [15]	BM	7.992489	5.336129
ALL_44	POSITIVE	RELAPSE	46XY	BM	10.89946	5.738832
ALL_45	NEGATIVE	DIAGNOSIS	NA	PBL	10.84403	6.22847
ALL_46	NEGATIVE	DIAGNOSIS	46 XY [18]	BM	7.179438	4.968322
ALL_47	POSITIVE	DIAGNOSIS	NA	BM	9.956452	5.30653
ALL_48	NEGATIVE	DIAGNOSIS	47XYY	PBL	9.380877	5.286138
ALL_49	NEGATIVE	DIAGNOSIS	46XX	BM	9.466752	5.346818
ALL_50	NEGATIVE	RELAPSE	46,XY,t(2;16)(p15;q22)[5]/46,XY[25]	BM	9.362621	5.233042
ALL_51	NEGATIVE	DIAGNOSIS	46XY [15]; t(2;16)(p15;q22) [5]	BM	10.67229	6.100287
ALL_52	POSITIVE	DIAGNOSIS	46XX[2]/46XX,t(9;22)[3]	BM	7.999699	4.714951
ALL_53	POSITIVE	RELAPSE	46XYdup(2)(p21;q37)t(9;22)(q34;q11)[20]	BM	10.412	5.658134
ALL_54	POSITIVE	DIAGNOSIS	45XY,-7,t(9;22)(q34;q11)[20/20]	BM	8.997415	4.76714
ALL_55	NEGATIVE	RELAPSE	46XY [14]	BM	10.80116	5.7076
ALL_56	NEGATIVE	DIAGNOSIS	NA	BM	9.01478	5.295629
ALL_57	NEGATIVE	DIAGNOSIS	46 XY	BM	9.512726	5.108504
ALL_58	NEGATIVE	DIAGNOSIS	48,XY,t(2;2)(p21;p25),+5,i(6p),+8,i(9q)[11/25]	BM	9.822683	5.436239

BM= bone marrow blood; PBL= Peripheral blood

Table S2. Patient’s characteristic ex vivo AZD-1775 treatment in single agent or in combination

Sample ID	BCR-ABL1 translocation	Stage of the disease	Sample type	Karyotype
ALL #1	Negative	Relapse	BM	46,XY,t(12;16)(p15;q22) (3/30)
ALL #2	Negative	Relapse	BM	NA
ALL #3	Negative	Diagnosis	BM	47,XY,+10,add(16)(p13)[8]/47,XY,id.,del(7)(q11)[1]/N[1]
ALL #4	Negative	Relapse	BM	NA
ALL #5	Negative	Diagnosis	PBL	NA
ALL #6	Negative	Relapse	PBL	47,XX,t(1;2)(p22;q11),t(1;5)(p12;q13),i(7)(q10),der(4),der(11)t(4;11)(q21;q23),t(13;17)(q12;p11),+M(5/5)
ALL #7	Negative	Diagnosis	PBL	46,XY,dup(1)(q21q32),t(8;14)(q24;q32),del(7)(q32q36),add(11)(q23)(11)/46,XY,t(8;14),del(7q),add(11q)(5)/46,XY,t(8;14),del(7q)(2)/46,XY,dup(1q),t(8;14),del(7q),add(11q),add(12)(q24)(1)
ALL #8	Negative	Relapse	BM	NA
ALL #9	Positive	Relapse	BM	NA
ALL #10	Positive	Diagnosis	BM	46,XX,t(8;12)(q22;p13),t(9;22)(q34;q11)(1)/47,XX,t(8;12),t(9;22),-random,+2Ms(3)/48,XX,+8,t(8;12),t(9;22),+M(1)/46,XX,t(8;12),t(9;22),i(17)(q10)(1)/46,XX,t(8;12),i(8)(q10),t(9;22)(1)/N(13)
ALL #11	Positive	Diagnosis	PBL	del(9q) 34%/ +Ph 57,5%
ALL #12	Positive	Diagnosis	BM	NA
ALL #13	Positive	Diagnosis	BM	49,XY,+der(3)del(3)(p11),+4,+der(5)del(4)(p11),t(9;22)(q34;q11)
ALL #14	Negative	Diagnosis	BM	NA
ALL #15	Negative	Relapse	BM	NA

BM= bone marrow blood; PBL= Peripheral blood

Table S3. Quantitative analyses of G2/M checkpoint related genes. Differential gene expression of 24 genes involved in the regulation of the G2/M checkpoint of primary leukemic cells in comparison to normal mononuclear cells (MNCs). In the table the primary leukemic samples have been divided into three groups based on the ex vivo sensitivity to AZD-1775. Very Good IC50<5uM; Good IC50< 10uM; poor IC50>10 uM.

PRIMARY LEUKEMIC CELLS Vs MNCS	Sample	Mean Cq	Mean Efficiency Corrected Cq	Normalized Expression	Relative Normalized Expression	Regulation	P-Value
PKMYT1	VERY GOOD	29.43	29.43	0.00844	64.35907	64.35907	0.004867
GADD45A	VERY GOOD	22.43	22.59	0.96603	2.80771	2.80771	0.011876
CHEK1	VERY GOOD	25.09	24.17	0.32371	11.11864	11.11864	0.023908
GADD45B	POOR	25.81	25.81	0.32260	0.15870	-6.30132	0.027860
BRCA1	VERY GOOD	24.46	23.74	0.43507	1.64114	1.64114	0.053765
GADD45B	VERY GOOD	25.02	25.02	0.17943	0.08827	-11.32884	0.059560
ATR	VERY GOOD	25.08	23.98	0.36950	0.26836	-3.72638	0.068452
CDC25C	VERY GOOD	28.07	27.86	0.02502	10.27334	10.27334	0.083565
MAPK14	VERY GOOD	23.48	23.48	0.52360	0.40793	-2.45138	0.088226
UBC	GOOD	21.75	22.98	3.14030	0.26203	-3.81642	0.119868

CCNA1	GOOD	31.29	31.96	0.00621	3.26626	3.26626	0.135636
MAPK14	GOOD	25.92	25.92	0.41082	0.32007	-3.12430	0.137909
CHEK2	VERY GOOD	26.69	25.12	0.16764	0.55001	-1.81815	0.148619
CCNB2	VERY GOOD	24.08	23.55	0.49537	12.80125	12.80125	0.151765
CCNA2	VERY GOOD	24.75	24.39	0.27842	9.53094	9.53094	0.170384
CDK1	VERY GOOD	24.28	23.21	0.62784	16.36977	16.36977	0.176841
ATR	GOOD	26.46	25.30	0.63040	0.45784	-2.18415	0.178589
CCNB1	VERY GOOD	24.85	24.85	0.20258	9.46808	9.46808	0.194237
GADD45A	GOOD	23.27	23.43	2.29611	6.67353	6.67353	0.209029
WEE1	VERY GOOD	24.79	24.24	0.30714	3.19940	3.19940	0.213592
UBC	VERY GOOD	18.92	19.99	5.86028	0.48898	-2.04507	0.228821
GADD45B	GOOD	25.26	25.26	0.64689	0.31823	-3.14240	0.229593
CDK1	GOOD	27.24	26.04	0.37720	9.83468	9.83468	0.265804
CCNB2	GOOD	27.22	26.62	0.25174	6.50538	6.50538	0.268660
CHEK1	GOOD	28.63	27.58	0.12917	4.43660	4.43660	0.269676
CCNB1	GOOD	27.70	27.70	0.11963	5.59111	5.59111	0.279290
CDK1	POOR	27.73	26.51	0.19749	5.14910	5.14910	0.280883
CCNA2	GOOD	27.88	27.48	0.13926	4.76718	4.76718	0.283551
CDC25C	GOOD	31.63	31.40	0.00920	3.77543	3.77543	0.295813
WEE1	GOOD	27.03	26.44	0.28635	2.98288	2.98288	0.300946
ATM	GOOD	26.60	24.83	0.87250	0.09139	-10.94255	0.323136
TP53	POOR	24.94	24.22	0.97140	1.93351	1.93351	0.323960
ATM	VERY GOOD	23.59	22.02	1.43716	0.15053	-6.64324	0.346950
CCNB1	POOR	28.07	28.07	0.06735	3.14780	3.14780	0.354245
CHEK1	POOR	28.79	27.74	0.08456	2.90427	2.90427	0.370479
CCNB2	POOR	28.14	27.52	0.09816	2.53668	2.53668	0.373012
CCNA2	POOR	28.40	27.99	0.07095	2.42884	2.42884	0.373430
CDC25B	GOOD	27.06	27.64	0.12461	0.12801	-7.81166	0.386695
CDC25B	VERY GOOD	24.53	25.05	0.17552	0.18032	-5.54562	0.395532
CHEK2	POOR	28.12	26.46	0.20455	0.67114	-1.49000	0.417725
WEE1	POOR	27.30	26.71	0.17272	1.79916	1.79916	0.424973
ATM	POOR	23.76	22.18	3.97147	0.41597	-2.40399	0.426138
ATR	POOR	25.47	24.35	0.88497	0.64274	-1.55584	0.433466
GADD45A	POOR	25.24	25.42	0.42226	1.22729	1.22729	0.443490
PLK3	GOOD	31.66	32.34	0.00478	0.05154	-19.40330	0.468920
UBB	GOOD	22.89	22.22	5.31015	0.51387	-1.94603	0.471815
PLK3	VERY GOOD	27.83	28.42	0.01697	0.18289	-5.46764	0.483102
PKMYT1	GOOD	36.59	36.59	0.00025	1.92270	1.92270	0.491260
FANCD2	POOR	26.98	26.39	0.21457	0.66706	-1.49911	0.552795
CDC25B	POOR	25.33	25.88	0.30714	0.31554	-3.16917	0.556587
CDKN1A	GOOD	28.07	27.25	0.16264	0.73573	-1.35919	0.581715
TP53	VERY GOOD	23.12	22.45	1.06836	2.12649	2.12649	0.586743
FANCD2	VERY GOOD	25.07	24.52	0.25317	0.78707	-1.27053	0.607733
UBB	POOR	21.60	20.97	9.21530	0.89177	-1.12137	0.609863
BRCA1	GOOD	27.60	26.79	0.22389	0.84456	-1.18404	0.640770
BLM	GOOD	28.14	27.32	0.15515	1.13687	1.13687	0.675464
CDC25C	POOR	32.91	32.68	0.00276	1.13328	1.13328	0.681660
FANCD2	GOOD	27.55	26.94	0.20127	0.62572	-1.59815	0.693531
CCNA1	VERY GOOD	30.98	31.65	0.00182	0.95544	-1.04664	0.706258
CHEK2	GOOD	28.22	26.56	0.26308	0.86316	-1.15854	0.715387
CDKN1A	POOR	26.71	25.93	0.29519	1.33538	1.33538	0.733341
PLK3	POOR	27.66	28.25	0.05928	0.63904	-1.56485	0.745289
CDKN1A	VERY GOOD	25.45	24.70	0.22347	1.01093	1.01093	0.748896
PKMYT1	POOR	36.23	36.23	0.00024	1.79545	1.79545	0.772607
UBC	POOR	20.40	21.55	6.14821	0.51300	-1.94930	0.837465

UBB	VERY GOOD	19.78	19.20	10.14642	0.98187	-1.01846	0.840311
TP53	GOOD	26.42	25.65	0.49368	0.98263	-1.01768	0.849969
CCNA1	POOR	32.43	33.12	0.00202	1.06395	1.06395	0.873590
BLM	POOR	27.71	26.90	0.15095	1.10605	1.10605	0.894410
MAPK14	POOR	24.75	24.75	0.67259	0.52401	-1.90834	0.906429
BLM	VERY GOOD	25.73	24.98	0.18447	1.35169	1.35169	0.911966
BRCA1	POOR	27.39	26.59	0.18722	0.70622	-1.41600	0.956251
