# Supplementary figures and images for: Targeting WEE1 to enhance conventional therapies for acute lymphoblastic leukemia
Source: J Hematol Oncol. 2018 Aug 1;11:99. doi: 10.1186/s13045-018-0641-1 (PMC6090987; doi:10.1186/s13045-018-0641-1)

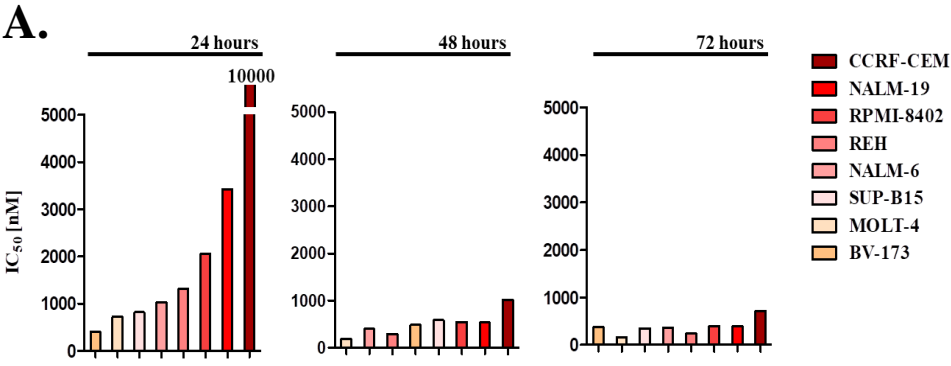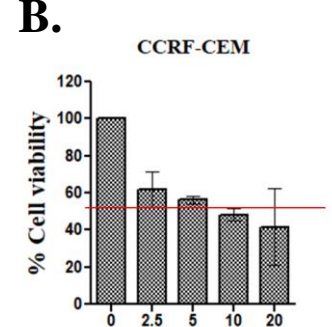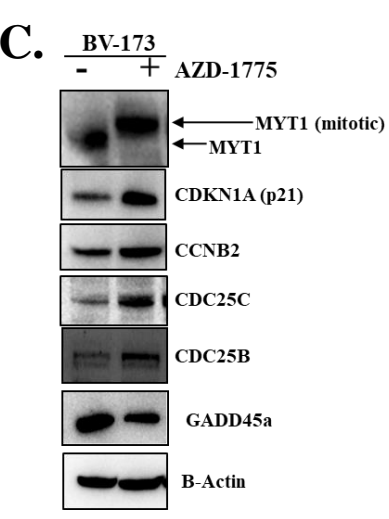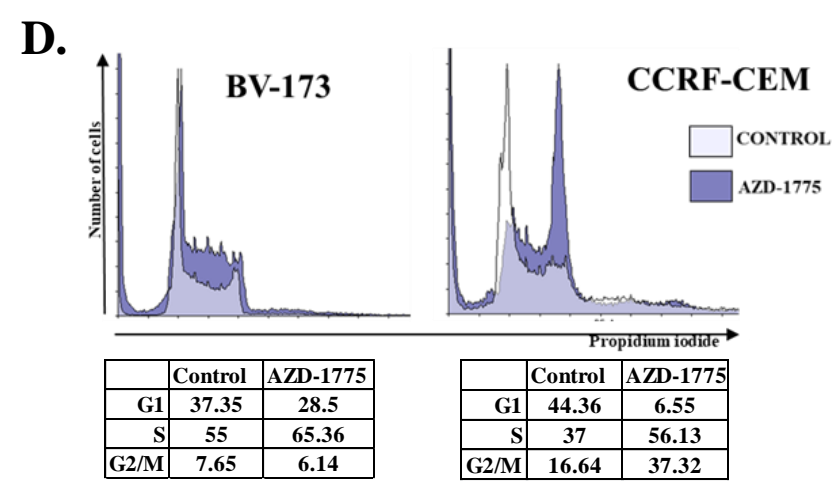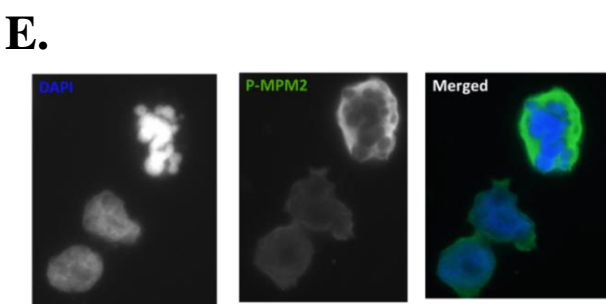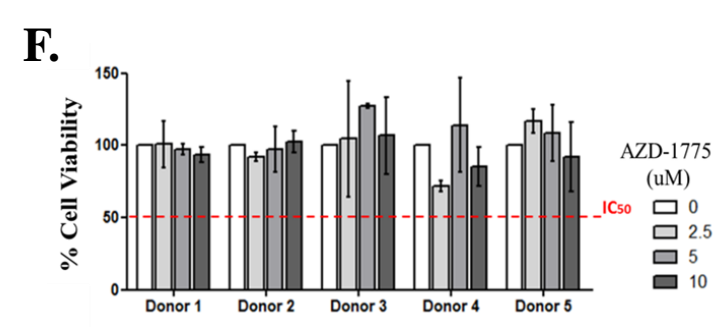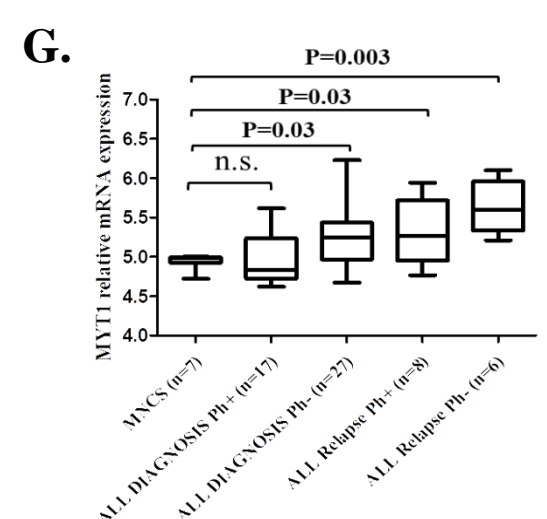

**A.**

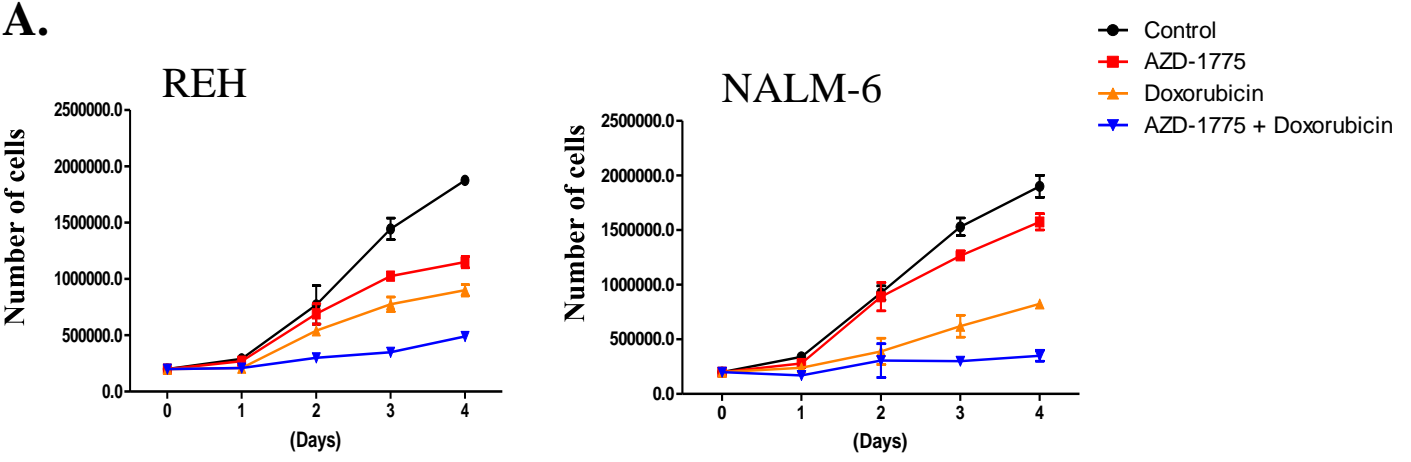

**B.**

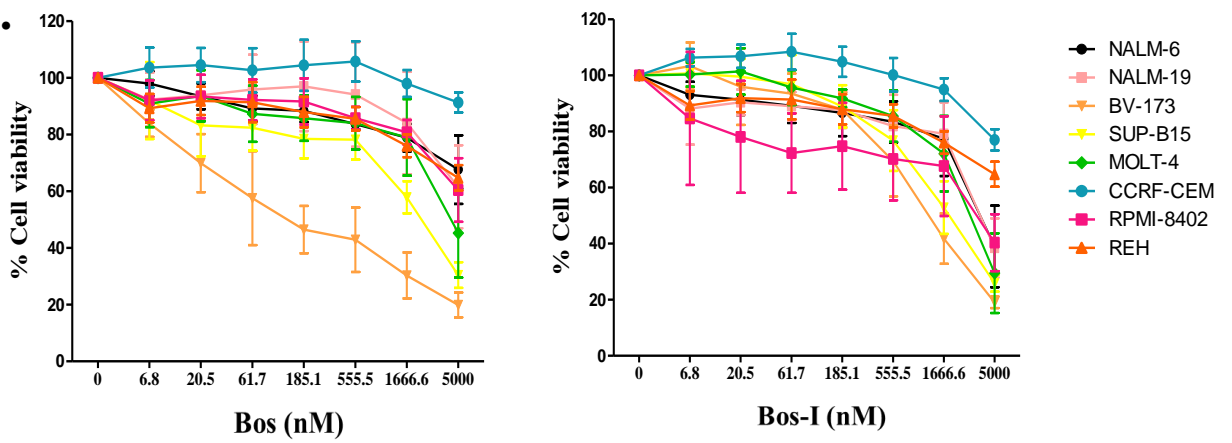

**C.**

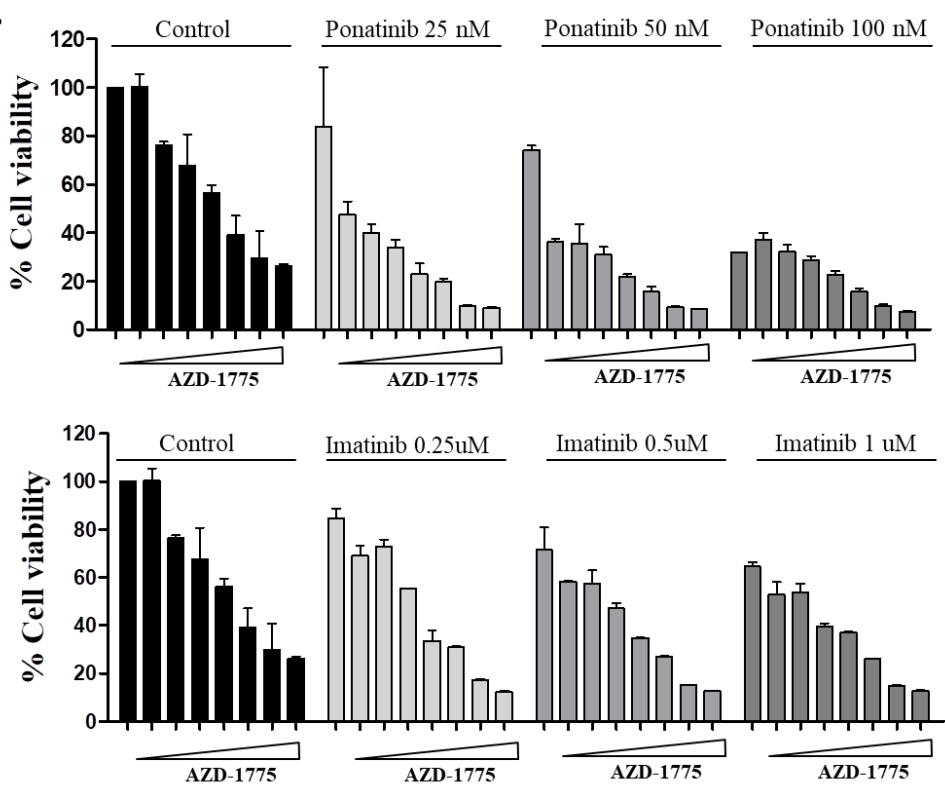

A.

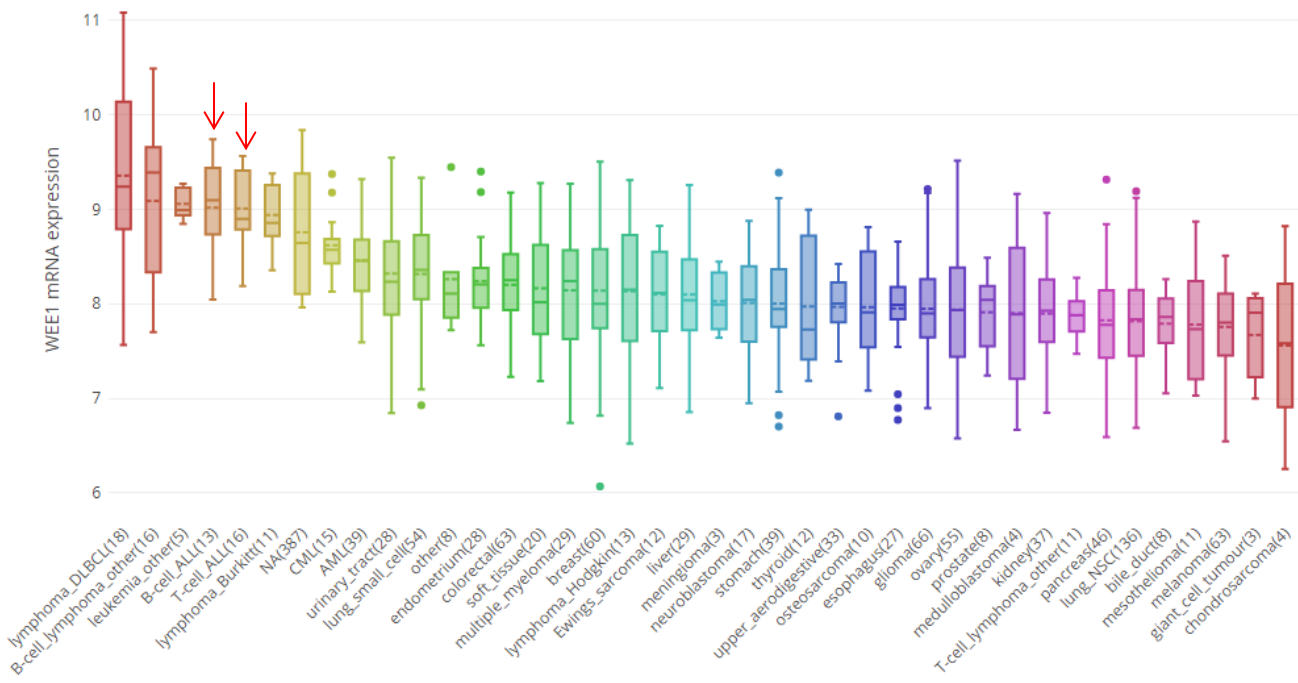

Supplement: Supplementary file 2 — Figure S1. Efficacy of AZD-1775 used as single agent. A) The graph shows the IC50 values of B/T-ALL cell lines treated with AZD-1775 for 24, 48, and 72 h. B) Cell viability analysis on CCRF-CEM cell lines showing the effect of high doses of AZD-1775. The percentage of viable cells is depicted relative to untreated controls. C) Immunoblot analysis on BV-173 treated with AZD-1775 (IC50) for 12 h. D) Cell cycle analysis in BV-173 and CCRF-CEM cell lines treated with AZD-1775 (IC50) for 12 h. E) Immunofluorescence analysis of BV-173 cells treated with AZD-1775 (IC50) for 12 h and, then, stained with DAPI and phospho-MPM2. In the picture, a cell dying in mitosis is reported with apoptotic bodies strongly positive for phospho-MPM2 antibody. Representative images are shown at × 100 magnification. F) Viability of mononuclear cells isolated from the peripheral blood of 5 healthy donors incubated with increasing concentration of AZD-1775 (2.5, 5, and 10 uM) for 24 h. G) MYT1 transcript levels in samples isolated from adult BCR-ABL1-positive ALL at diagnosis (n = 17), adult BCR-ABL1-negative ALL at diagnosis (n = 27), adult BCR-ABL1-positive ALL at relapse (unpaired, n = 8), adult BCR-ABL1-negative ALL at relapse (unpaired, n = 6), and in MNCs (n = 7) from the peripheral blood of healthy donors. One-way ANOVA test was performed to assess statistical significance. Results are expressed as Log10 2 exp.[−(ΔΔCt). Figure S2. AZD-1775 in combination with chemotherapy agents and tyrosine kinase inhibitors. A) Growth curve of BV-173 and REH cell lines treated for 4 days with AZD-1775 (185 nM) and doxorubicin (25 nM). B) Viability analyses in ALL cell lines incubated for 24 h with Bos or Bos-I (6 to 5000 nM). The percentage of viable cells is depicted relative to untreated controls. C) Cell viability analysis of BV-173 cell line treated with AZD-1775 (6 to 5000 nM, dilution rate 1:3) and with ponatinib (25, 50, 100 nM) or imatinib (250, 500, and 1000 nM) for 24 h. The percentage of via [file 13045_2018_641_MOESM2_ESM.pdf]
